# Oncolytic adenovirus delivery of neoantigens sensitizes low-mutation tumors to anti-PD-1 therapy and prevents metastasis

**DOI:** 10.1038/s41392-025-02511-5

**Published:** 2025-12-23

**Authors:** Ke-Yu Shen, Shi-Zhe Yu, Ying-Han Su, Sun-Zhe Xie, Chen Zhang, Hao Xu, SamI Yang, Tian-Tian Zou, Yan Fu, Hao Wang, Lin Fang, Yan Zheng, Chang-Qing Su, Lun-Xiu Qin

**Affiliations:** 1 Hepatobiliary Surgery, Department of General Surgery, Huashan Hospital & Cancer Metastasis Institute, Fudan University, Shanghai, China; 2 Institutes of Biomedical Sciences, Fudan University, Shanghai, China; 3 Center of Interventional Radiology and Vascular Surgery, Department of Radiology, Zhongda Hospital, Medical School, Southeast University, Nanjing, China; 4 Jiangsu Center for the Collaboration and Innovation of Cancer Biotherapy, Cancer Institute, Xuzhou Medical University, Xuzhou, Jiangsu China; 5 Department of Pancreatic Surgery, Shanghai General Hospital & Shanghai Jiao Tong University School of Medicine, Shanghai, China; 6 National Center for Liver Cancer (NCLC), Navy Medical University, Shanghai, China

**Keywords:** Drug development, Vaccines

## Abstract

Neoantigen vaccines and oncolytic viruses are emerging immunotherapies that can reshape the tumor microenvironment (TME). However, tumors with low mutation burdens often respond poorly to immunotherapies because of their limited immunogenicity. Developing effective immunotherapy strategies for these types of tumors remains a significant challenge. In this study, we engineered oncolytic adenoviruses to accurately amplify neoantigen expression within tumor cells, which demonstrated superior efficacy compared to synthetic long peptide vaccines and showed enhanced effectiveness in a low mutation burden intrahepatic cholangiocarcinoma model. Building on this, we further developed NeoViron, which coexpresses neoantigens and Flt3L, a dendritic cell growth factor, to promote antigen presentation and T-cell infiltration simultaneously. NeoViron significantly inhibited tumor growth and prevented metastasis in intrahepatic cholangiocarcinoma animal models. Mechanistically, NeoViron enhanced the cytotoxicity of CD8+ T cells and promoted the expansion of CD69+ CD8+ tissue-resident memory T cells and TCF1+ CD8+ stem-like T cells to promote anti-tumor immunity and immune memory. When combined with anti-PD-1, it further enhances the cytotoxicity of tissue-resident memory T cells to eradicate solid tumors. These findings demonstrate that NeoViron can effectively sensitize low-mutation tumors to immunotherapy by increasing neoantigen expression and antigen-presentation efficacy, offering a promising strategy for cancer treatment, particularly for tumors with scarce neoantigens.

## Introduction

Tumor neoantigens, arising predominantly from somatic mutations, represent tumor-specific epitopes that are recognized as nonself by the host immune system. These mutation-derived antigens are presented via MHC molecules and can activate specific T-cell responses. A key advantage of neoantigens over tumor-associated antigens is their absence in normal tissues, evading central immune tolerance and minimizing the off-target effects on healthy tissues.^1^ This unique property has positioned neoantigen-based vaccines as a promising therapeutic approach for personalized cancer immunotherapy. Current vaccine platforms employing synthetic long peptide (SLP), mRNA, and DNA-based vaccines^2^ have demonstrated clinical efficacy in advanced malignancies with high tumor mutational burdens (TMBs), including melanoma,^3^ non-small cell lung cancer^4^ and colorectal cancer.^5^ However, their therapeutic potential remains limited in low-TMB cancers such as cholangiocarcinoma (CCA) and pancreatic cancer, where insufficient neoantigen diversity and suboptimal antigen presentation compromise both neoantigen vaccine efficacy and immune checkpoint inhibitor responses.^6–8^ In addition, impaired antigen presentation pathways, the exclusion of T cells by the tumor immune barrier, and T cell exhaustion represent significant factors contributing to the failure of neoantigen-based therapies.^2^

Oncolytic viruses (OVs) offer a multifaceted therapeutic strategy by selectively replicating in tumor cells to induce immunogenic cell death or lyse cells. These characteristics enable oncolytic viruses to accurately deliver therapeutic payloads to mediate transgene amplification and reprogram tumor immune microenvironment. Currently, various OV platforms, including adenovirus, herpes simplex virus (HSV), vaccinia virus, and Newcastle disease virus (NDV), have demonstrated distinct mechanisms of tumor targeting and immune activation. Several clinically approved OVs, such as Oncorine (oncolytic adenovirus, OAV) and T-VEC (oncolytic HSV), have validated the feasibility of virotherapy, with over 100 candidates currently in clinical development.^9^ VG161, an engineered oncolytic HSV designed to express IL-12, IL-15, IL-15Rα, and a PD-1-PD-L1 blocking fusion protein, demonstrated promising outcomes in a multicenter phase 1 trial involving patients with advanced hepatocellular carcinoma. The results showed that VG161 was well-tolerated with no observed dose-limiting toxicities. By remodeling the tumor immune microenvironment, it not only reversed resistance to systemic therapy but also enhanced therapeutic efficacy in patients previously sensitive to checkpoint inhibitors.^10^ In another clinical study, intravenous administration of NDV-GT, a recombinant NDV carrying a porcine α1,3GT gene, achieved a disease control rate of 90% in patients with relapsed/refractory metastatic cancer. Notably, this oncolytic virus introduces the xenogeneic antigen α-galactosidase, which effectively kills tumor cells by inducing a hyperacute rejection response.^11^ Those two recently reported clinical studies highlighted the significant potential of oncolytic viruses in treating liver cancers.

Growing evidence shows that increasing tumor antigen expression or introducing xenogeneic antigens can enhance tumor immunogenicity to improve immunotherapy efficacy. The proximity amplification and tagging of cytotoxic haptens (PATCH) technology, activated by red light or ultrasound, can enhance T-cell activation and cytotoxicity by promoting the aggregation and amplification of synthetic antigen-fluorescein on the surface of tumor cells.^12^ Meanwhile, oncolytic viruses have also been engineered to deliver CD19 into tumor cells, thereby rendering the tumors susceptible to targeting by CD19-specific CAR-T cells.^13^ OVs can also redirect preexisting T-cell immunity by delivering epitopes from viruses, e.g., lymphocytic choriomeningitis virus or SARS-CoV-2, to tumor sites, enabling tumor-irrelevant bystander T cells to recognize viral epitope-expressing malignancies.^14^ However, since these strategies introduce shared antigens, unlabeled or uninfected tumor cells may evade immune attack, posing a risk of immune escape. This raises an intriguing possibility: Is it feasible to engineer oncolytic viruses to amplify the expression levels of neoantigens within tumor cells, simultaneously enhancing tumor immunogenicity and inducing an immune memory response that can also target uninfected tumor cells, particularly in low-TMB cancers?

In our previous studies of a set of OAVs, we engineered a genetic switch by fusing the HIF-1α oxygen-dependent degradation domain (ODD) to the E1a protein, thereby restricting viral replication selectively in the hypoxic tumor microenvironment. Additionally, we enhanced the infection efficiency across multiple cancers by incorporating a chimeric Ad5/Ad11 fiber knob.^15^ Therefore, we hypothesized that OAV-mediated neoantigen delivery could overcome the limitations of conventional immunotherapies in low-TMB tumors. To validate this concept, we engineered NeoViron, a multifunctional OAV platform incorporating two synergistic components: (1) tandem minigenes encoding validated neoantigens from murine tumor models, and (2) murine Fms-like tyrosine kinase 3 ligand (Flt3L), a dendritic cell (DC) growth factor known to expand intratumoral conventional type 1 dendritic cells (cDC1s) critical for antigen cross-presentation.^16^ NeoViron is designed to synergize with four complementary antitumor mechanisms. First, direct delivery of neoantigens targeted to tumor cells enhances their immunogenic visibility. Second, viral oncolysis generates an in situ antigen reservoir through tumor cell lysis. Third, Flt3L expression promotes cDC1 expansion to potentiate antigen presentation. Finally, virus-induced inflammation may remodel the immunosuppressive tumor microenvironment (TME). This combinatorial approach positions NeoViron as a versatile platform for personalized therapy across solid tumors, with particular promise for low-TMB malignancies refractory to existing immunotherapies. Furthermore, its compatibility with immune checkpoint inhibitors such as PD-1 antibodies suggests broad potential for therapeutic synergy.

## Results

### Oncolytic virus-mediated neoantigen delivery elicits potent tumor-specific immunity across multiple tumor models

To establish a proof-of-concept for neoantigen-directed oncolytic virotherapy, we first characterized mutational landscapes in four syngeneic C57BL/6 mouse tumor models, including melanoma B16F10, colon cancer MC38, hepatocellular carcinoma (HCC) Hep53.4, and intrahepatic cholangiocarcinoma (ICC) mICCN-4. Specifically, mICCN-4 is a mouse cell line driven by Akt and the Notch intracellular domain (NICD), which are key characteristics of ICCs (Supplementary Fig. 1a–c).^17^

**Fig. 1:**
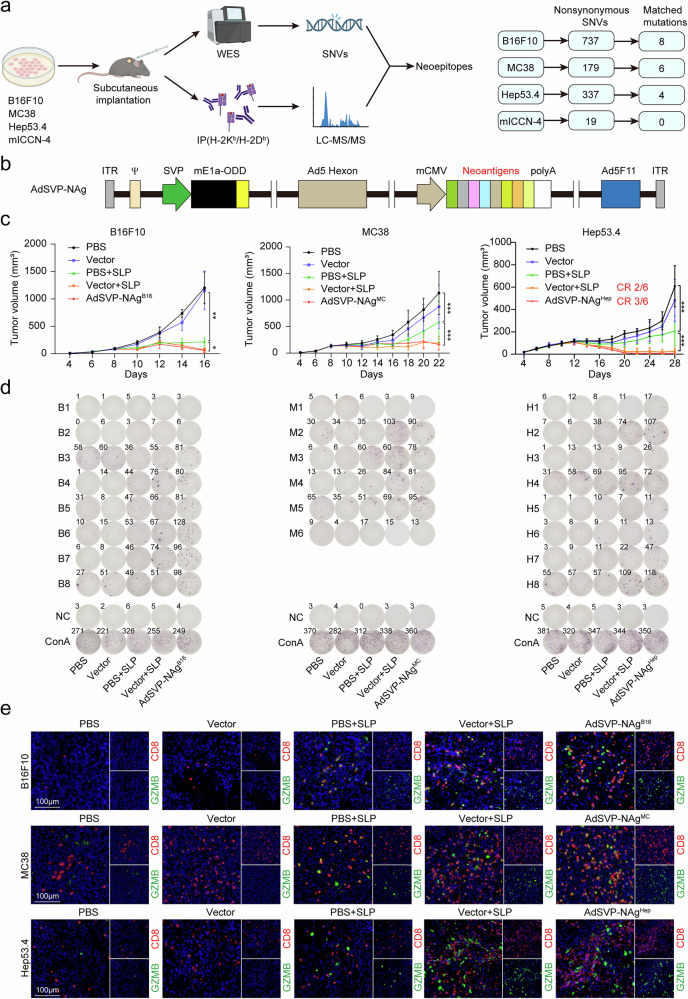
Neoantigen identification and delivery via oncolytic adenovirus vectors. **a** Schematic representation of neoantigen identification combining WES and MS. This figure was created via resources from BioRender (www.biorender.com). **b** Design of the neoantigen delivery system. The expression cassette of neoantigen cDNA sequences corresponding to the 29-mer long peptides covering the mutated epitopes was inserted into the E3 region of the survivin promoter (SVP)-regulated oncolytic adenovirus vector Ad5SVPF11 to generate a novel OAV, AdSVP-NAg. C57BL/6 mice inoculated with B16F10 melanoma, MC38 colon carcinoma, or Hep53.4 hepatocellular carcinoma cells were treated with PBS, vector (Ad5SVPF11), SLP, vector combined with SLP, or AdSVP-NAg. Tumor growth curves (**c**) (*n* = 6 mice per group), representative images (**d**) of the IFN-γ ELISpot of neoantigen-specific T cells from the spleen in each group and immunofluorescence images (**e**) of tumor tissues. The data are shown as the means ± SDs (**c**) and are representative of two independent experiments (**c**–**e**). Significance was calculated by two-way ANOVA (**c**). *P < 0.05, **P < 0.01, ***P < 0.001

To increase the specificity and reliability of neoantigen identification, we integrated whole-exome sequencing (WES) with liquid chromatography‒tandem mass spectrometry (LC‒MS/MS) for MHC-I immunopeptidome analysis to detect tumor neoantigens.^18^ WES identified 737, 179, 337, and 19 nonsynonymous single nucleotide variants (SNVs) in B16F10, MC38, Hep53.4, and mICCN-4 tumors, respectively. Among these, MS-validated neoantigens included 8 epitopes in B16F10 (B1–B8), 6 in MC38 (M1–M6), and 8 (from 4 SNVs) in Hep53.4 (H1–H8) (Supplementary Table 1), whereas mICCN-4 exhibited no MS-detectable neoantigens due to subthreshold expression (Fig. 1a and Supplementary Fig. 1d–f). Notably, the B16F10-derived B8 epitope (AALTFRRL) matched a previously reported Ndufs6 mutation.^19^ NetMHCpan 4.1 predictions confirmed the MHC-I binding affinities of the identified epitopes (Supplementary Table 1).

We tandemly linked the gene sequences corresponding to the 29-mer extended epitopes of each tumor and loaded them into the E3 region of the previously described Ad5SVPF11 vector,^15^ naming the generated OAV AdSVP-NAg (AdSVP-NAg^B16^ for B16F10; AdSVP-NAg^MC^ for MC38; AdSVP-NAg^Hep^ for Hep53.4; AdSVP-NAg as an umbrella term for all three) (Fig. 1b). Viral tropism assessment via AdSVP-DsRed (Supplementary Fig. 1g) demonstrated selective tumor cell replication of the Ad5SVPF11 vector in four tumor cell lines (Supplementary Fig. 1h). AdSVP-DsRed-mediated E1a expression at 96 h after infection increased 10^^^4-fold in the tumor cell lines compared with 200-fold in the AML12 hepatocytes (Supplementary Fig. 1i). The delivery efficiency of the 3 corresponding cell lines B16F10, MC38, and Hep53.4 was also detected (Supplementary Fig. 1j). These results demonstrated that Ad5SVPF11 has high tropism for tumors and can effectively deliver payloads.

In vivo efficacy tests using the subcutaneous implantation models of B16F10, MC38, and Hep53.4 cells in C57BL/6 mice revealed that intratumoral AdSVP-NAg administration achieved tumor regression comparable to that of SLP vaccination combined with intratumoral injections of empty vector (Fig. 1c). Notably, AdSVP-NAg^Hep^ induced early complete response (CR) in 50% (3/6) of Hep53.4 tumors, whereas the combination of SLP with the empty vector achieved 33.33% (2/6) early CR (Fig. 1c and Supplementary Fig. 2a).

ELISpot assays revealed that both AdSVP-NAg and the combination of SLP and vector elicited broader neoantigen-specific T-cell responses, with the number of T cells specific for multiple neoantigens (B4, B5, B6, B7, B8, M2, M3, M4, H2, H4, H7, H8) significantly increasing (Fig. 1d and Supplementary Fig. 2b–d). OAV infection and enhanced intratumoral infiltration of GZMB+ CD8+ cytotoxic T lymphocytes were detected via immunohistochemistry (IHC) of E1A and immunofluorescence of GZMB and CD8 (Fig. 1e and Supplementary Fig. 2e, f). Together, these data demonstrated that OAV-mediated neoantigen delivery elicits potent tumor-specific immunity across multiple tumor models, underscoring its dual mechanism of direct oncolysis and adaptive immune activation.

### Oncolytic virus delivery could overcome neoantigen scarcity in immunologically cold cholangiocarcinoma

**Fig. 2:**
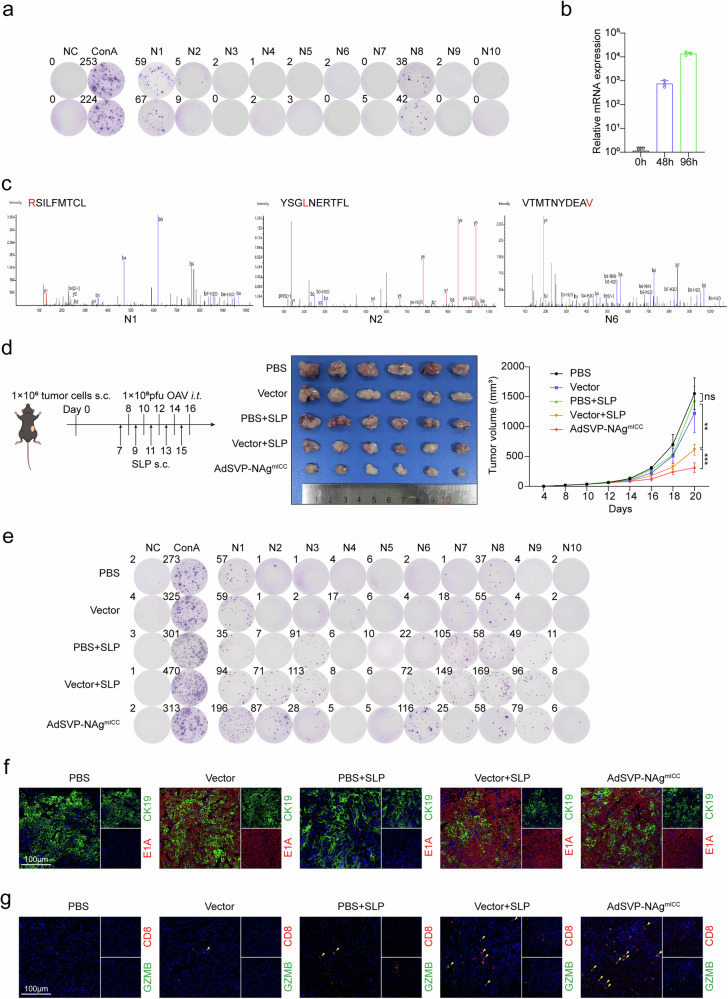
Efficacy of AdSVP-NAgmICC in ICCs with few mutations. **a** Representative images of IFN-γ ELISpots of neoantigen-specific T cells from the spleens of C57BL/6 mice inoculated with mICCN-4. **b** Relative expression of neoantigen (NAg) in mICCN-4 cells infected with AdSVP-NAg^mICC^ at 0 h, 48 h, and 96 h determined by qRT‒PCR. **c** Mass spectra of epitopes in mICCN-4 cells detected by LC‒MS/MS following infection with AdSVP-NAg^mICC^ for 96 h. **d**‒**g** C57BL/6 mice inoculated with mICCN-4 were treated with PBS, vector, SLP, vector combined with SLP, or AdSVP-NAg^mICC^. **d** Left, experimental schematic. On Day 0, 1 × 10⁶ tumor cells were resuspended in 100 μL of saline and subcutaneously injected into the mice. Intratumoral injections of PBS or 2 × 10⁸ pfu OAV were administered every other day starting from Day 8, while subcutaneous injections of SLP were initiated on Day 7 and continued every other day for a total of five doses. Middle, tumor images at the end point. Right, tumor growth curves (*n* = 6 mice per group). **e** Representative images of IFN-γ ELISpots of neoantigen-specific T cells from the spleens of each group. **f**, **g** Representative immunofluorescence images of tumor tissues. The data are shown as the means ± SDs (**d**) and are representative of two independent experiments (**a**–**g**). Significance was calculated by two-way ANOVA (**c**). **P < 0.01, ***P < 0.001. ns not significant

Given that only 19 nonsynonymous SNVs were detected in the murine ICC cell line mICCN-4 through WES and that no neoantigen epitopes were identified by MS, we employed the NetMHCpan algorithm to predict the mutation-derived neoantigen epitopes, and 10 candidate epitopes of 7 SNVs with affinities to MHC-I were selected (Supplementary Table 2). Two epitopes identified by the ELISpot assay could induce specific immune responses in treatment-free tumor-bearing mice (Fig. 2a). As OVs can promote the presentation of tumor-irrelevant epitopes by tumor cells, thereby redirecting bystander T cells to target tumor cells,^14^ AdSVP-NAg^mICC^ was constructed with these 10 epitopes, and we further evaluated whether it can increase the expression and presentation of neoantigens in ICC cells to promote immune recognition and destruction of the tumor. After 96 h of infection, AdSVP-NAg^mICC^ delivery of neoantigens was successfully detected via qPCR, and three of the upregulated epitopes (N1, N2, and N6) were detected via LC‒MS/MS (Fig. 2b, c).

Moreover, AdSVP-NAg^mICC^ more significantly inhibited in vivo tumor growth than the combination of SLP and vector (Fig. 2d and Supplementary Fig. 3a). This contrasts with the comparable efficacy observed in the B16F10, MC38, and Hep53.4 tumor models mentioned above (Fig. 1c), demonstrating that delivering neoantigens has greater advantages in low-mutation tumors. ELISpot assays revealed that the neoantigen-specific T-cell response for N1, N2, N6, and N9 was significantly enhanced after AdSVP-NAg^mICC^ treatment (Fig. 2e and Supplementary Fig. 3b), with viral replication confirmed by CK19+ tumor cell-specific E1A expression detected by immunofluorescence staining (Fig. 2f). The increased infiltration of GZMB+ CD8+ T cells suggested enhanced cytotoxic function of CD8+ T cells in the AdSVP-NAg^mICC^ group (Fig. 2g and Supplementary Fig. 3c). However, SLP alone did not significantly inhibit tumor growth even though it also induced a neoantigen-specific T-cell response.

These data indicate that OAV-mediated neoantigen amplification can facilitate more efficient recognition and elimination of neoantigen-expressing cells by the immune system to overcome the immune evasion mechanisms of low-mutational burden tumors such as ICCs.

### Flt3L coexpression in oncolytic viruses further potentiates dendritic cell-mediated antitumor immunity and enhances its antitumor effects

To amplify antigen presentation, we engineered AdSVP-Flt3L to secrete the DC growth factor Flt3L (Supplementary Fig. 4a). The expression and secretion of Flt3L from mICCN-4 or Hep53.4 cells infected with AdSVP-Flt3L were determined by qPCR and ELISA (Supplementary Fig. 4b, c).

**Fig. 3:**
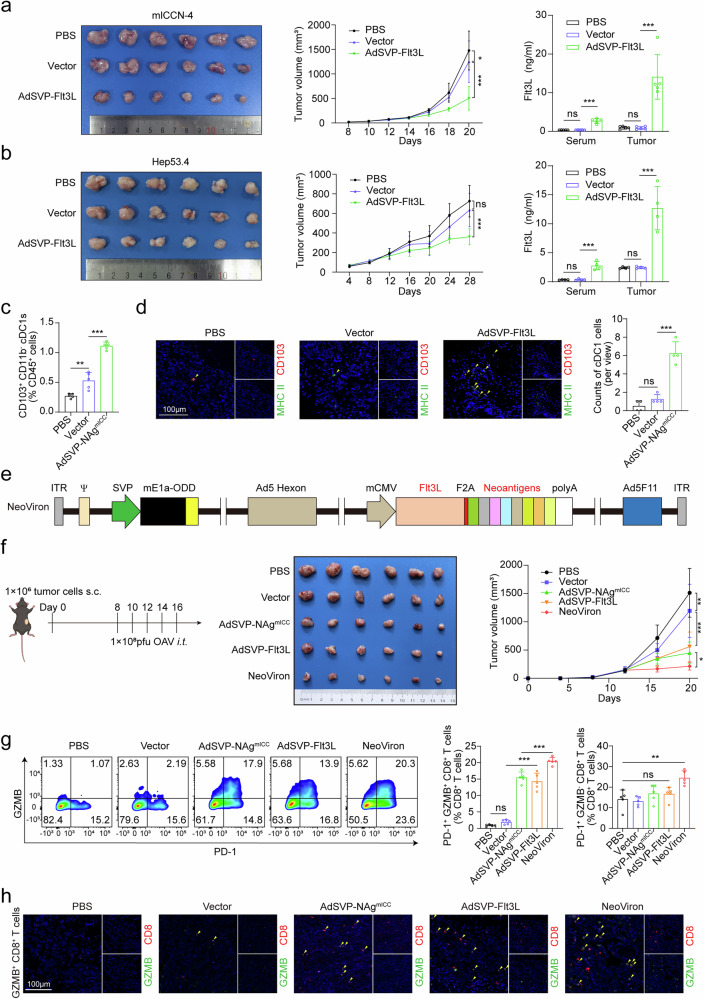
Construction and in vivo validation of NeoViron. **a**‒**d** C57BL/6 mice inoculated with mICCN-4 or Hep53.4 cells were treated with PBS, vector or AdSVP-Flt3L. For the mICCN-4 model, treatment was initiated on day 8, with intratumoral injections of 1 × 10⁸ pfu OAV administered every other day for a total of five doses. For Hep53.4 tumors, treatment commenced on Day 6 with intratumoral injections of 2 × 10⁸ pfu OAV every other day for five doses. **a**, **b** Left, tumor images at the end point. Middle, tumor growth curves (*n* = 6 mice per group). **c**, **d** CD103+ cDC1s were examined by flow cytometry (**c**) or immunofluorescence staining (MHC II+ CD103+ cells) (**d**). **e** Design of NeoViron, coexpressing neoantigens and Flt3L. **f**–**h** C57BL/6 mice inoculated with mICCN-4 were treated with PBS or 1 × 10⁸ pfu OAV (vector, AdSVP-NAg^mICC^, AdSVP-Flt3L or NeoViron) from Day 8 every other day for a total of five doses. **f** Left, experimental schematic. Middle, tumor images at the end point. Right, tumor growth curves (*n* = 6 mice per group). **g** Percentages of PD1+ GZMB+ CD8+ T cells and PD1+ GZMB- CD8+ T cells infiltrating the tumor tissues of each group. **h** Representative immunofluorescence images of GZMB+ CD8+ T cells in tumor tissues. The data are shown as the means ± SDs (**a**–**g**) and are representative of two (**a**–**h**) independent experiments. Significance was calculated via one-way ANOVA (**a**–**c**, **g**) or two-way ANOVA (**a**, **b**, **f**). *P < 0.05, **P < 0.01, ***P < 0.001. ns not significant

To explore the effects of secreted Flt3L on DC cells, we cocultured mouse bone marrow-derived DC cells with conditioned media from AdSVP-Flt3L-infected cells and found that the conditioned media significantly promoted the in vitro proliferation of DC cells in a concentration-dependent manner, particularly favoring their differentiation into cDC1s (Supplementary Fig. 4d–f). Moreover, intratumoral injections of AdSVP-Flt3L significantly inhibited in vivo tumor growth and increased the levels of Flt3L in both the blood and tumor tissues of the mice in which mICCN-4 and Hep53.4 cells were subcutaneously implanted (Fig. 3a, b and Supplementary Fig. 4g). Flow cytometry and immunofluorescence assays revealed a marked increase in the intratumoral DC/cDC1 population (Fig. 3c, d and Supplementary Fig. 4h–j).

Building on the promising results from AdSVP-NAg^mICC^ and AdSVP-Flt3L, we developed a novel OAV, NeoViron, designed to coexpress Flt3L and neoantigens separated by F2A cleavage (Fig. 3e). Tail vein injection of NeoViron did not cause obvious adverse effects or infect major organs such as the liver, lungs, or kidneys (Supplementary Fig. 4k). Compared with AdSVP-NAg^mICC^ or AdSVP-Flt3L alone, intratumoral injection of NeoViron induced greater tumor inhibition (Fig. 3f and Supplementary Fig. 4i) and significantly enhanced the intratumor infiltration of CD8+ T cells, particularly the proportion of PD-1+ GZMB+ CD8+ T cells, as well as the DC/cDC1 populations detected by flow cytometry and immunofluorescence staining (Fig. 3g, h and Supplementary Fig. 5a–i). Additionally, in vivo CD8+ cell depletion abrogated the tumor control effect mediated by NeoViron (Supplementary Fig. 5j–n). These findings indicate that NeoViron synergizes with cDC1 recruitment to enhance CD8+ T-cell cytotoxicity, resulting in superior tumor control to that of monotherapies.

### Synergistic immune checkpoint blockade unlocks exhausted T-cell potential to increase the efficacy of NeoViron

**Fig. 4:**
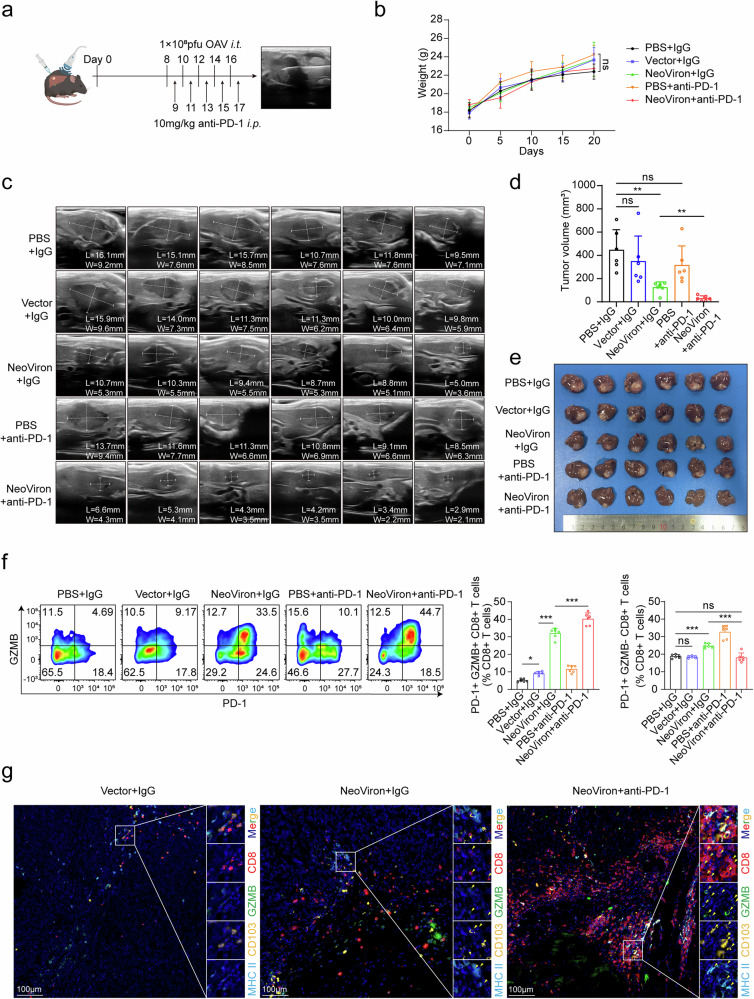
NeoViron exhibits synergistic effects with anti-PD-1. **a**‒**g** C57BL/6 mice with livers orthotopically implanted with mICCN-4 were administered intratumoral injections under ultrasound guidance starting from Day 8, with either PBS, a 1 × 10⁸ pfu vector, or NeoViron every other day for five doses. Concurrently, intraperitoneal injections of 10 mg/kg anti-PD-1 were initiated on day 9 and administered every other day for five doses. Experimental schematic (**a**), body weight curves of mice during treatment (**b**), ultrasound images (W, width; L, length) (**c**), tumor volumes (volume = W^2^ × L × 0.52) (**d**), and tumor images (**e**) within liver tissues at the end point. **f** Percentages of PD1+ GZMB+ CD8+ T cells and PD1+ GZMB- CD8+ T cells infiltrating the tumor tissues of each group. **g** Multiplex immunofluorescence images showing the colocalization of GZMB+ CD8+ T cells and CD103+ MHC II+ cDC1s. The data are shown as the means ± SDs (**b**, **d**, **f**) and are representative of two independent experiments (**b**–**g**). Significance was calculated via one-way ANOVA (**d**, **f**) or two-way ANOVA (**b**). *P < 0.05, **P < 0.01, ***P < 0.001. ns not significant

In addition to enhancing the recruitment of DC/cDC1 and PD-1+ GZMB+ CD8+ T cells, NeoViron also induced a certain increase in PD-1+ GZMB- CD8+ T cells, which is an indicator of potential T-cell exhaustion (Fig. 3g). Hence, the combination of NeoViron with anti-PD-1 therapy may further increase the efficacy of treatment. As expected, compared with NeoViron alone, the combination of NeoViron with anti-PD-1 significantly inhibited tumor growth in mICCN-4 subcutaneous implantation models (Supplementary Fig. 6a–c). To better understand its possible influence on the tumor immune microenvironment, we administered NeoViron via ultrasound-guided intratumoral injections in orthotopic implantation model mice, which is a potential clinical therapeutic modality (Fig. 4a). This combination reduced the size of orthotopic ICC tumors with a synergy coefficient of 1.16 and prolonged the survival of the mice without any weight loss or obvious adverse effects (Fig. 4b–e and Supplementary Fig. 6d).

Furthermore, the combination treatment significantly promoted the infiltration of GZMB+ PD-1+ cytotoxic CD8+ T cells compared with NeoViron monotherapy (Fig. 4f). Consistently, this combination also extended the infiltration of DCs and cDC1s (Supplementary Fig. 6e, f). Multiplex immunofluorescence (mIHC) revealed coordinated cDC1-CD8+ T-cell interactions within tumor niches (Fig. 4g and Supplementary Fig. 6g). These results indicate that anti-PD-1 therapy can further increase the cytotoxicity of neoantigen-specific T cells induced by NeoViron and that their combination has a synergetic effect on solid tumors such as ICCs with few mutations.

### Combination therapy promotes the clonal expansion of CD69+ CD8+ Trm and TCF1+ CD8+ Tstem cells

**Fig. 5:**
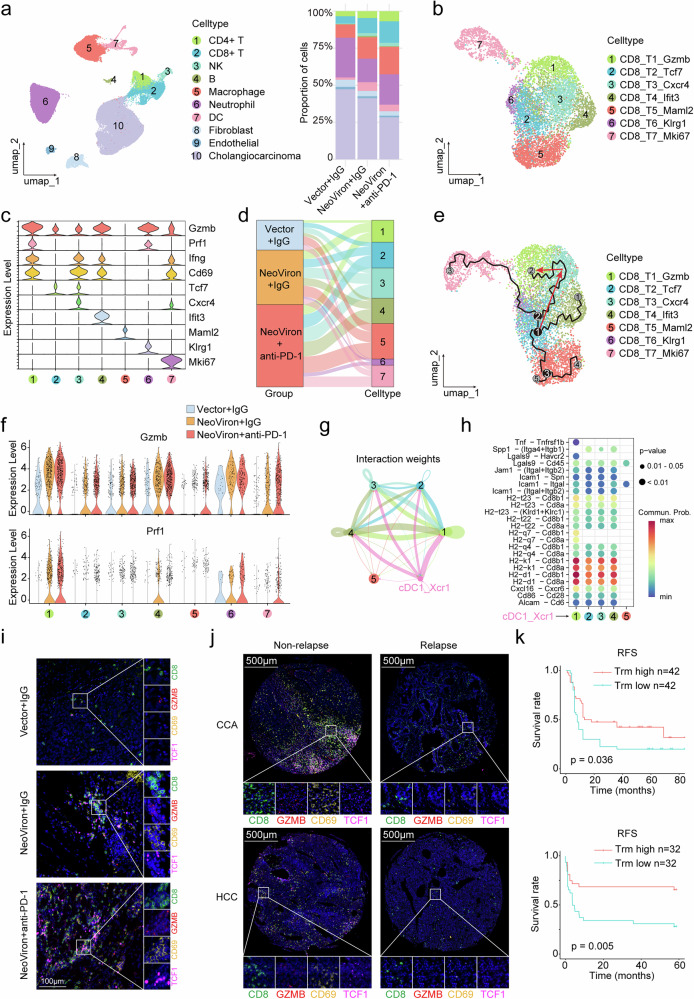
Combination therapy promotes the expansion of CD69+ CD8+ Trm and TCF1+ CD8+ Tstem cells. **a** Left, UMAP visualization of cell clusters from six samples across three groups: Vector plus IgG, NeoViron plus IgG, and NeoViron plus anti-PD-1. Right, bar plot showing the proportions of different cell clusters in each group. **b** UMAP visualization of CD8+ T-cell subsets. **c** Violin plots representing the expression levels of marker genes for each CD8+ T-cell subset. **d** Alluvial plot showing the amounts of each CD8+ T-cell subset across different groups. **e** Pseudotime trajectory analysis of CD8+ T-cell subsets generated by Monocle 3. **f** Violin plots representing the expression levels of Gzmb and Prf1 in each CD8+ T-cell subset across different groups. **g** Circle plot depicting the interaction weights between cDC1s and each CD8+ T-cell subset generated by CellChat. **h** Dot plot of ligand‒receptor interactions between cDC1s and each CD8+ T-cell subset. **i** Representative multiplex immunofluorescence images showing tumor-infiltrating CD69+ CD8+ Trm and TCF1+ CD8+ Tstem cells in different groups. **j** Representative multiplex immunofluorescence images showing the CD8+ Trm and Tstem cell infiltration levels in the ICC and HCC cohorts. **k** RFS curves of the Trm high and low groups. Log-rank Mantel‒Cox test

To further elucidate the underlying mechanism, we performed single-cell RNA sequencing (scRNA-seq) on NeoViron-treated tumors. Dimensionality reduction and clustering of the scRNA-seq data revealed a total of 10 cell populations, among which the numbers of CD4+ T cells, CD8+ T cells, and DCs were increased in the combination treatment group (Fig. 5a and Supplementary Fig. 7a). The CD4+ T cells and DCs were further clustered into four subpopulations: CD4_T1_Ifng, CD4_T2_Il13, CD4_T3_Foxp3, and CD4_T4_Tcf7; cDC1_Xcr1, cDC2_CD209a, cDC3_Ccr7, and pDC_Siglech, respectively. Consistent with the above flow cytometry results, the numbers of IFN-γ+ CD4+ T cells and cDC1s were elevated following the combination treatment (Supplementary Fig. 7b, c).

CD8+ T cells were clustered into seven subpopulations, including CD8_T1_Gzmb, characterized by highly expressed cytotoxic molecules such as Gzmb, Prf1, and Ifng, as well as the residency marker Cd69, which is consistent with tissue-resident memory T cells (Trm); CD8_T2_Tcf7, characterized by the expression of the stemness-related transcription factor Tcf7, representing stem-like T (Tstem) cells; CD8_T3_Cxcr4, coexpressing Cd69 and Tcf7, possibly representing an intermediate state between T1 and T2; CD8_T4_Ifit3, characterized by high expression of interferon response-related genes; CD8_T5_Maml2, which does not express any effector molecules, likely representing the resting T cells; CD8_T6_Klrg1, with high levels of cytotoxic molecules but Cd69-negative, accounting for a minor proportion; and CD8_T7_Mki67, representing proliferative T cells, characterized by high expression of Mki67 (Fig. 5b, c). The intratumoral infiltration of CD8_T1_Gzmb, CD8_T2_Tcf7 and CD8_T3_Cxcr4 was the most significantly increased following the combination treatment (Fig. 5d). Monocle3 pseudotime analysis revealed the differentiation trajectories from CD8_T2_Tcf7 to other CD8 + T-cell subpopulations, which was consistent with its stem-like characteristics (Fig. 5e). We focused on the trajectory from CD8_T2_Tcf7 through CD8_T3_Cxcr4 to CD8_T1_Gzmb. Along this trajectory, the expression of Cd69, Gzmb, and Prf1 increased during this transformation, whereas Tcf7 expression decreased (Supplementary Fig. 7d). CD8_T1_Gzmb presented the highest expression of the cytotoxic molecules Gzmb and Prf1 among all the subpopulations, which significantly increased following NeoViron treatment, and its cytotoxicity was further increased by anti-PD-1 therapy (Fig. 5f). Intercellular communication analysis revealed stronger interactions between cDC1s and CD8_T1_Gzmb, which were predominantly mediated by the MHC-I signaling pathway (Fig. 5g, h and Supplementary Fig. 7e). Furthermore, the increased intratumoral infiltration of CD69+ CD8+ Trm cells and TCF1+ CD8+ Tstem cells after NeoViron and anti-PD-1 treatment was further validated by immunofluorescence (Fig. 5i). In summary, scRNA-seq of NeoViron-treated tumors revealed the clonal expansion of stem-like TCF1+ CD8+ T cells and their differentiation into Trm (CD8_T1_Gzmb) subsets.

Postoperative samples from HCC and CCA patients were collected to investigate the associations between these two cell populations and clinical outcomes. We conducted a quantitative analysis of the infiltrating CD8+ T cells, Trm cells, and Tstem cells in tumors and grouped the patients according to the median cutoff value to compare overall survival (OS) and relapse-free survival (RFS) between the two groups (Fig. 5j, k and Supplementary Fig. 8a, b). The results revealed no significant difference in prognosis between patients with high and low CD8+ T-cell infiltration in tumors. However, patients with high Trm infiltration presented significantly longer OS and RFS than did those with low Trm infiltration. Furthermore, patients with high Tstem infiltration tended to have longer OS and RFS than did those with low Tstem infiltration (Fig. 5j, k and Supplementary Fig. 8a, b). Additionally, we utilized transcriptomic data from CCA and HCC patients in the TCGA database to classify patients into high- and low-level groups on the basis of the signature genes of each T-cell subtype, namely, CD8+ T cells (CD8+ T cells (CD8), Trm (CD8, CD69, GZMB), and Tstem (CD8, TCF7), and conducted survival analysis. HCC patients with high Trm infiltration characteristics had longer OS and RFS than did those with low infiltration characteristics. However, owing to the limited sample size, no significant difference was observed between the two groups of CCA patients (Supplementary Fig. 8c, d).

Taken together, these results demonstrate that NeoViron promotes the expansion of TCF1+ CD8+ Tstem cells and CD69+ CD8+ Trm cells. TCF1+ CD8+ T stem cells can differentiate into various CD8+ T-cell subtypes, particularly CD69+ CD8+ Trm cells, which exhibit the strongest cytotoxic activity among all subtypes. The results from clinical samples also suggest a potential long-term protective effect of NeoViron, suggesting the translational potential of NeoViron.

### NeoViron protects CD69+ CD8+ Trm cells against tumor metastasis

**Fig. 6:**
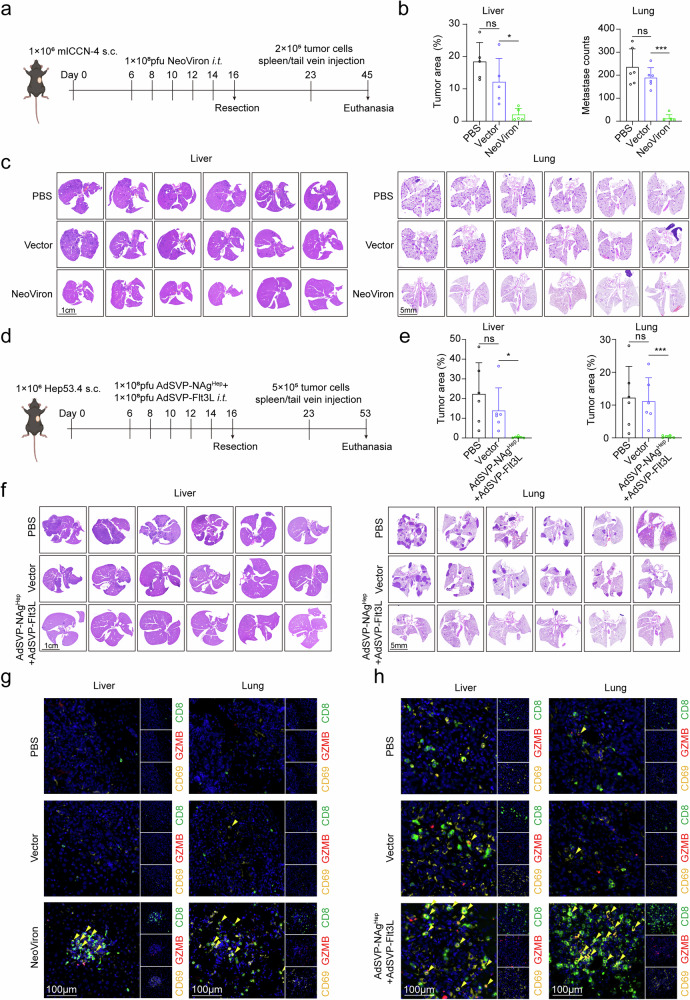
NeoViron-induced CD8+ Trm cells prevent tumor metastasis. **a**‒**g** C57BL/6 mice inoculated with mICCN-4 or Hep53.4 cells were treated with PBS, vector or NeoViron/AdSVP-NAg^Hep^+ AdSVP-Flt3L 5 times, followed by tumor excision on day 16. One week later, liver/lung metastasis models were established by injecting tumor cells via the spleen/tail vein. **a**, **d** Experimental schematic. **b**, **e** Tumor area of liver metastases and counts of lung metastases analyzed by ImageJ. **c**, **f** HE-stained images of liver and lung metastases at the end of the experiment. **g** Multiplex immunofluorescence images showing CD8+ Trm cells in liver and lung metastases. The data are shown as the means ± SDs (**b**, **e**) and are representative of two independent experiments (**a**–**h**). Significance was calculated via the Kruskal‒Wallis test (**b**, **e**). *P < 0.05, ***P < 0.001. ns not significant

To further evaluate the protective effects of the NeoViron-induced adaptive immune response, we established a mouse model for preventing tumor metastasis. Five intratumoral injections of NeoViron were administered after the establishment of subcutaneous mICCN-4 tumors, followed by tumor excision. One week later, the mice were rechallenged with mICCN-4 tumor cells via splenic capsule injection or tail vein injection. After 3 weeks, the mice were euthanized, and the liver or lungs were collected to assess metastasis (Fig. 6a). HE staining results demonstrated that NeoViron significantly reduced the tumor size of liver metastases and the number of lung metastatic foci (Fig. 6b, c). In addition to ICC, AdSVP-NAg^Hep^ plus AdSVP-Flt3L also exhibited preventive effects against HCC metastasis (Fig. 6d–f). Immunofluorescence staining revealed clustering of CD69+ CD8+ Trm cells in the liver and lung metastatic foci from the NeoViron or AdSVP-NAg^Hep^ plus AdSVP-Flt3L treatment groups in both the mICCN-4 and Hep53.4 models (Fig. 6g, h). These results indicate that NeoViron-induced CD8+ Trm cells are positively correlated with prevention of metastasis.

## Discussion

The paucity of immunogenic neoantigens in solid tumors creates a critical barrier to effective immunotherapy. Our study revealed that OAV-mediated neoantigen delivery (NeoViron) overcomes this limitation through three synergistic mechanisms: (1) amplification of subdominant neoantigens to immunogenic levels, (2) Flt3L-driven expansion of cDC1 antigen-presenting cells, and (3) generation of CD8+ cytotoxic Trm and self-renewing Tstem cell populations (Supplementary Fig. 9). This multimodal approach achieved 50% CR in immunogenic HCC tumors and converted “cold” ICC microenvironments into T-cell-inflamed niches, demonstrating broad applicability across mutationally diverse cancers.

The precise identification and efficient delivery of neoantigens redefine therapeutic boundaries. Our integrated WES/MS pipeline was proven to be effective, as most of the identified neoantigens successfully triggered specific T-cell responses. However, MS has failed to identify neoantigens in mutation-scarce murine ICC cell lines, possibly because their expression levels are under the detection threshold of this technology. Consequently, we shifted to predicting neoantigens from WES, and several predicted neoantigens were also capable of eliciting T-cell responses.

The current mainstream vaccine platforms, such as SLP and mRNA vaccines, are designed primarily to deliver neoantigens to lymph nodes. However, insufficient lymph node targeting and low delivery efficiency remain challenging for these platforms. More critically, they fail to overcome the suppressive immune barriers of tumors and are usually ineffective against “immune-desert” tumors lacking neoantigens.^2^ In contrast, viral vectors can deliver effector molecules to cancer cells to redirect their phenotype and reshape the immune microenvironment. For example, the delivery of the transcription factors PU.1, IRF8, and BATF3 to cancer cells via replication-deficient adenovirus enables the direct reprogramming of cancer cells into dendritic cells.^20^ Compared with replication-deficient adenovirus vectors, OVs have the unique capability of selectively and directly delivering therapeutic payloads to cancer cells and increasing transgene copy number and expression efficiency via virus replication. Chen et al. developed a strategy using OV that delivers tumor-irrelevant epitopes to tumor cells, effectively redirecting bystander T cells to target tumor cells.^14^ Similarly, introducing the porcine α1,3GT gene into NDV triggers a hyperacute rejection response against tumors, resulting in an impressive 90% disease control rate in 20 patients with relapsed/refractory metastatic cancer.^11^ On the basis of this concept, we developed AdSVP-NAg, which is based on an OAV vector designed to directly deliver neoantigens to tumor cells, thereby increasing their immunogenicity. Compared with SLP, AdSVP-NAg exhibited significantly superior tumor control in neoantigen-rich tumors, including B16F10, MC38, and Hep53.4 tumors. However, its efficacy was comparable to that of the simple combination of SLP and the OV. We propose that this is because the immunogenicity of neoantigen-rich tumors is already sufficient to induce neoantigen-specific T-cell-mediated cytotoxicity. The lack of increased antigen-presenting efficacy has limited the ability of AdSVP-NAg to further increase its immunogenicity. In contrast, in the neoantigen-deficient ICC model mICCN-4, AdSVP-NAg exhibited far superior efficacy compared with the combination of SLP and the vector. These findings suggest that the delivery of neoantigens via our OAV may offer greater advantages in treating neoantigen-deficient tumors such as CCA and pancreatic cancer, as mentioned above.

Multiple studies have identified CD8+ Trm cells and CD8+ T stem cells as key contributors to the efficacy of neoantigen vaccines,^21–24^ and their presence is closely linked to patients’ response to immune checkpoint inhibitors.^25,26^ CD8+ Trm cells reside in peripheral tissues such as the lungs, intestines, liver, and tumor sites, where they demonstrate potent tumor-killing capabilities and possess memory properties.^27,28^ In contrast, CD8+ T cells have limited cytotoxicity but possess self-renewal capacity and the ability to differentiate into cytotoxic CD8+ T cells, particularly in the context of immunotherapy.^29,30^ In the present study, NeoViron significantly increased the intratumoral abundance of CD69+ CD8+ Trm cells and TCF1+ CD8+ Tstem cells while also revealing the differentiation trajectory from TCF1+ CD8+ Tstem cells to CD69+ CD8+ Trm cells. Notably, anti-PD-1 therapy further enhanced the self-renewal capacity of CD8+ T stem cells and upregulated the expression of effector molecules (e.g., GzmB and Prf1) in CD69+ CD8+ Trm cells. Furthermore, analysis of postoperative patient samples revealed that patients without tumor metastases presented higher levels of CD8+ Trm and Tstem cell infiltration in their primary tumors than did those who developed metastases. In mouse metastasis models, NeoViron was shown to effectively prevent liver and lung metastases. These findings suggest that NeoViron, either as monotherapy or in combination with anti-PD-1 therapy, represents a promising clinical strategy for activating antitumor immunity and preventing metastasis.

Since our primary focus was on delivering neoantigens to tumor cells, we considered only MHC-I-restricted neoantigens derived from nonsynonymous mutations that can be presented by tumor cells. Indeed, mutation-independent pathways, such as aberrant transcription, translation, and posttranslational events, can also serve as potential sources of neoantigens.^2,31^ Neoantigens derived from chemically induced RNA alternative splicing or IFN-γ-induced ribosome slippage have also been identified as potential targets for immunotherapy.^32,33^ In addition, MHC class II-restricted neoantigens play important roles in the antitumor response.^34,35^ Therefore, more efforts are warranted to investigate whether the delivery of a broader spectrum and variety of neoantigens via oncolytic viruses can further improve therapeutic efficacy.

In conclusion, by engineering an oncolytic virus that concurrently (1) amplifies tumor neoantigens, (2) recruits cDC1 antigen-presenting cells, and (3) generates T-cell memory, NeoViron has established a new paradigm for overcoming immunotherapy resistance in low-TMB tumors. Its synergy with PD-1 blockade and capacity to prevent metastatic recurrence position this platform as a transformative approach for personalized cancer immunotherapy, particularly in hepatobiliary cancers with limited treatment options.

## Materials and methods

### Cell culture

C57BL/6 mice derived from the melanoma cell line B16F10, the colon cancer cell line MC38, the hepatocellular carcinoma cell line Hep53.4, and the normal hepatocyte cell line AML12 were obtained from the Shanghai Cell Bank and the Chinese Academy of Science (Shanghai, China).

The murine ICC cell line mICCN-4 was isolated from a cholangiocarcinoma model established via murine Akt/NICD hydrodynamic injection. Specifically, 20 μg of NICD, 4 μg of Akt, and 2 μg of Sleeping Beauty transposase (SB) plasmids were dissolved in 2 mL of saline and injected into 6-week-old C57BL/6 mice via hydrodynamic tail vein injection (HTVI) within 5 s. Six weeks later, the tumor tissues were harvested and subjected to subcutaneous transplantation for further purification. Once the subcutaneous tumors reached 10 mm × 10 mm, the tumor tissues were excised, and the cell line was extracted.

All the cell lines were maintained in Dulbecco’s modified Eagle’s medium (DMEM) (Gibco, USA) supplemented with 10% fetal bovine serum (FBS) (Gibco, USA) and 1% penicillin/streptomycin (Beyotime, China) in a humidified incubator containing 5% CO_2_ at 37 °C.

### Synthetic peptides

Synthetic peptides representing neoantigens for ELISpot assays and vaccination were synthesized by GeneScript Biotechnology (USA). The sequences of the peptides are listed in Supplementary Table 3.

### SLP vaccination

The formulation of the SLP vaccine was adapted from previous studies,^36^ with each dose containing 20 μg of each 29-mer neoantigen peptide in Supplementary Table 3 and 50 μg of poly I:C (MedChemExpress, USA), dissolved in 200 μL of a 10% DMSO/90% PBS solution.

### Mouse models

Six-week-old male C57BL/6 mice were purchased from GemPharmatech Co., Ltd. (Nanjing, China). All the animal studies were approved by the Welfare and Ethics Group of the Laboratory Animal Science Department of Fudan University, with Approval Certificate No. 2023-HSYY-287-JZS, and the animal experimental procedures strictly complied with the ethical requirements and the ARRIVE guidelines 2.0. All the mice were housed under standard specific pathogen-free (SPF) conditions with a controlled temperature (22 ± 1 °C) and a 12/12-h light/dark cycle. The health status and welfare of the mice were monitored daily. Euthanasia was performed when the mice reached predefined humane endpoints, including signs of imminent mortality, severe physiological compromise, or tumor burden accompanied by ulceration and behavioral abnormalities.

For the subcutaneous tumor model, 2 × 10^5^ or 1 × 10^6^ tumor cells resuspended in 100 µL of physiological saline were injected subcutaneously into the axillary region of the mice.

For the orthotopic tumor transplantation model, when the subcutaneous mICCN-4 tumor reached ~500 mm³ in size, the tumor tissue was harvested and cut into 1 mm × 1 mm blocks. These tumor blocks were implanted under the liver capsules of the mice, and the liver wounds were sealed with surgical glue.

For the liver metastasis model, 2 × 10^5^ mICCN-4 or 5 × 10^5^ Hep53.4 cells resuspended in 200 µL of physiological saline were slowly injected into the spleens of C57BL/6 mice.

For the lung metastasis model, 2 × 10^5^ mICCN-4 or 5 × 10^5^ Hep53.4 cells resuspended in 200 µL of physiological saline were injected into the tail vein of C57BL/6 mice.

### Treatment of tumor-bearing mice

For subcutaneous tumors, the following treatments were used: intratumoral injection of 100 μL of PBS, vector, AdSVP-NAg, AdSVP-Flt3L or NeoViron; multipoint injection at seven positions (superior, inferior, left, right, anterior, central, posterior) around and within the tumor; subcutaneous injection of SLP; and intraperitoneal injection of a rat IgG isotype control (BioXcell, USA; BE0089) or anti-PD-1 antibody (BioXcell, USA; BE0146). The mice were euthanized by cervical dislocation to collect tumor tissues for subsequent analysis when the measured tumor volume reached 2000 mm³ or the maximum diameter reached 20 mm.

For the CD4/CD8 T-cell depletion experiments, the mice received intraperitoneal injections of 200 μg of anti-CD4 (Selleck, USA; A2101), anti-CD8 (Selleck, USA; A2102), or rat IgG isotype control (Selleck, USA; A2116) antibodies. The first injection was administered 2 days before initiation of the therapeutic treatment, with subsequent injections repeated every 3 days until study termination.

For orthotopic tumors, intratumoral injection of PBS, vector, or NeoViron was performed under the guidance of a small animal ultrasound system (KOLO SiliconWave 30, China).

### MHC peptide immunoprecipitation and enrichment

To enrich MHC-bound peptides, 1 × 10^8^ B16F10, MC38, Hep53.4, and mICCN-4 cells or 200 mg of subcutaneous tumor tissue were lysed via lysis buffer containing 0.5% CHAPS (EMD Milipore, USA; 220201) and protease inhibitors (Thermo Fisher, USA; A32963). H-2Kb/H-2Db-specific peptides from the lysate were immunoprecipitated via anti-mouse MHC-I antibodies (BioXcell, USA; BE0077) conjugated to CNBr-activated beads (Cytivia, USA; 17043001). Peptides were eluted from the antibody-bead complexes via elution buffer containing 28% acetonitrile and 0.1% trifluoroacetic acid. The enriched peptides were subsequently desalted via a C18 column (Thermo Fisher, USA; 89870) according to the manufacturer’s instructions and evaporated to dryness via a vacuum concentrator. Before mass spectrometry analysis, the dried peptides were reconstituted in 4% formic acid.

### Mass spectrometry (MS)

Peptide samples were analyzed via liquid chromatography‒tandem mass spectrometry (LC‒MS/MS) on a quadrupole Orbitrap Exploris 480 mass spectrometer (Thermo Fisher, USA) connected to an UltiMate 3000 RSLC nanoliquid chromatography system (Dionex, USA). Approximately 500 ng of peptide was loaded onto a chromatography column (150 μm inner diameter, 250 mm length, Jupiter 3 μm C18 300 Å) for separation via a gradient of 5–30% ACN-0.1% FA at a flow rate of 600 nL/min. Each full MS spectrum was acquired at a resolution of 120,000, with an injection time and spectrum acquisition using tandem mass spectrometry on the most abundant multicharged precursor ions, lasting up to a maximum of 3 s.

All mass spectrometry data were analyzed via the DeepNovo workflow of PEAKS Studio 11 for de novo peptide sequencing and searched against the *Mus musculus* SwissProt (2024_03) database. The peptide search parameters were set to no enzyme digestion, methionine oxidation (+15.99 Da), 15 ppm parent mass error tolerance, and 0.03 Da fragment mass error tolerance. The search results were further filtered using a 70% DeepNovo score and a 3% false discovery rate (FDR). Finally, the peptide list was refined by filtering for peptide lengths of 8–10 amino acids.

### Whole exome sequencing (WES)

DNA extracted from B16F10, MC38, Hep53.4, and mICCN-4 subcutaneous tumors and C57BL/6 mouse tail tissues was subjected to WES, which was conducted by the HaploX Genomics Center. The DNA was fragmented, end-repaired, ligated with sequencing adapters, and amplified via PCR and size selection. Whole-exome capture was then performed via the Agilent SureSelect Mouse All Exon V1 capture kit. After library quality control, different libraries were pooled on the basis of their effective concentration and the target data output requirements, followed by Illumina PE150 sequencing. The effective sequencing data were aligned to the mouse reference genome mm9 via BWA (http://bio-bwa.sourceforge.net), generating alignment results in the BAM format. Somatic SNPs and Indels were identified via VarScan2 and functionally annotated via ANNOVAR.

### Peptide affinity prediction

The binding affinity of 8–10 amino acid peptides generated from nonsynonymous mutations to H-2 Kb and H-2Db alleles was predicted via NetMHCpan 4.1. The binding threshold for weak binder was set at rank <2%, and that for strong binder (SB) was set at rank <0.5%. For mICCN-4, the 8–10-mer mutant peptides with a binding threshold set at rank <2% to H2-Kb or H2-Db were considered candidate neoantigens. Ten neoantigens meeting these criteria were selected for viral incorporation.

### Construction of virus vectors

The construction and production of the oncolytic adenovirus vector for neoantigen delivery were based on the previously described Ad5SVPF11 (vector) and Ad5SVPF11-DsRed (AdSVP-DsRed) vectors.^15^ The survivin promoter was synthesized and cloned upstream of the adenovirus serotype 5 (Ad5) E1a gene. A 12-bp sequence (cacgaggctggc) within the CR2 region of E1a was deleted to generate a mutant E1a (mE1a), and the oxygen-dependent degradation (ODD) domain sequence of hypoxia-inducible factor-1a (HIF-1a) was fused downstream of mE1a as a genetic switch. Concurrently, the coding sequences for both the E1b-55kD and E1b-19kD proteins in the Ad5 E1 region were deleted. Furthermore, the fiber knob domain of Ad5 was replaced with the corresponding fragment from Ad11.

To construct AdSVP-NAg, the cDNA sequences corresponding to the 29-mer-long peptides covering the mutated epitopes were inserted into the E3 region of the oncolytic adenovirus vector in the form of tandem minigenes. To construct AdSVP-Flt3L, the gene sequence of murine Flt3L was inserted into the E3 region. For the construction of NeoViron, both the tandem minigene and the murine Flt3L gene sequences were inserted into the E3 region, which is connected by the 2A peptide sequence from the foot-and-mouth disease virus (F2A). All viruses were amplified in HEK293 cells and purified through cesium chloride gradient centrifugation. The viral titers were measured via the 50% tissue culture infective dose (TCID50) method.

### Oncolytic virus infection experiments in vitro

The cells were cultured in 6-well plates and infected with vector, AdSVP-DsRed, AdSVP-NAg, AdSVP-Flt3L, or NeoViron at an MOI = 50 pfu/cell. At 0 h, 48 h, and 96 h, red fluorescence from DsRed was captured via a fluorescence microscope, and total RNA was collected for qRT‒PCR analysis to detect the expression of E1A, Flt3L, or tandem minigenes.

### cDNA synthesis and quantitative real-time PCR (qRT‒PCR)

Total RNA was extracted from the cell lines via an RNA extraction kit (Beyotime, China) and reverse-transcribed into cDNA via the Prime-Script RT Reagent Kit (TaKaRa, Japan). Quantitative real-time PCR (qRT‒PCR) was performed via TB Green Fast qPCR Mix (TaKaRa, Japan) on an ABI7500 system (Thermo Fisher, USA) following the manufacturer’s protocol. Gene expression levels were normalized to those of β-actin and expressed as the relative copy number for each gene.

### Flt3L quantification via enzyme-linked immunosorbent assay (ELISA)

ELISA for the measurement of Flt3L was carried out following the manufacturer’s instructions. To quantify Flt3L in the cell supernatant, the supernatant was collected after the cells were infected with AdSVP-Flt3L for ELISA analysis. To measure Flt3L levels in the blood, retro-orbital bleeding was performed on tumor-bearing mice to collect blood. The samples were centrifuged at 10,000 rpm for 10 min, and the resulting sera were used for ELISA analysis. For the measurement of Flt3L in tumor tissues, the mice were euthanized, and the tumors were excised. One hundred milligrams of tumor tissue were finely sliced and incubated at 37 °C in 200 µL of PBS for 2 h. The supernatants were then collected and analyzed via a mouse Flt3L ELISA Kit (Proteintech, USA).

### ELISpot

Tumor-bearing mouse spleens were ground via a syringe plunger, filtered through a 40-μm cell strainer, and erythrocytes were lysed with erythrocyte lysing solution. Freshly isolated splenic lymphocytes (2 × 10⁵ cells/well) were cocultured with each neoantigen peptide (10 ng/µL) in RPMI-1640 medium supplemented with 10% FBS and incubated in IFN-γ ELISpot plates (Mabtech, Sweden; 3321-4AST-2) for 18–24 h at 37 °C. Negative controls were resuspended in RPMI-1640 medium supplemented with 10% FBS. The positive control groups were stimulated with ConA (Invitrogen, USA; 00-4978-93, 1:500). IFN-γ-secreting T cells were then detected via IFN-γ ELISpot assays following the manufacturer’s protocol. The developed spots were quantified via an AID iSpot ELISpot reader (AID, Germany).

### Generation of murine bone marrow (BM)-derived DCs

The femurs and tibias were excised from the mice, and the skin and muscles were carefully removed. After both ends of the bones were cut, the BM cells were flushed out with RPMI medium. The cells were passed through a 70-μm filter and centrifuged at 400 × *g* for 5 min to pellet the cells. Red blood cells were lysed by resuspension in 3 mL of ammonium chloride-potassium lysis buffer for 3 min. The cells were then incubated at a density of 1.5 × 10^6^ cells/mL in RPMI medium containing 10% FBS. Subsequently, 20 ng/mL recombinant mouse Flt3L (PeproTech, USA; 250-31 L) or supernatants from AdSVP-Flt3L-infected tumor cells and 300 pg/mL mouse GM-CSF (PeproTech, USA; 315-03) were added to the culture. After 9 days of culture, nonadherent and loosely adherent cells were collected and gently washed with PBS for flow cytometry analysis.

### Flow cytometry

To detect T cells in tumors, the tumor tissues were dissociated into single-cell suspensions, followed by the addition of 1× cell activation cocktail (BioLegend, USA; 423301) and activation at 37 °C for 4–6 h. For Fc receptor blockade, anti-mouse CD16/32 (BioLegend, USA; 101320, 1:50) was added to the cells for 10 min at 4 °C. The following antibodies were subsequently used to target surface markers: BV510 anti-mouse CD45 (BioLegend, USA; 103138, 1:50), PE/Cy7-CD3 (BioLegend, USA; 100220, 1:100), FITC-CD8a (BioLegend, USA; 100706, 1:100), BV605-CD4 (BD Biosciences, USA; 563151, 1:100), BV421-CD279 (BioLegend, USA; 135217, 1:100), and Fixable Viability Dye eFluor 780 (eBioscience, USA; 65-0865-14, 1:100). The cells were incubated with antibodies at 4 °C for 30 min, followed by fixation and permeabilization with Fix/Perm Buffer (BioLegend, USA; 426803) for 20 min. Subsequently, intracellular staining was performed via the following antibodies: APC-GZMB (BioLegend, USA; 372203, 1:20) and PE-IFN-γ (BioLegend, USA; 505808, 1:100), followed by incubation at 4 °C for 30 min.

To assess DCs, BM-derived DCs or tumor tissue cell suspensions were stained with the following antibodies targeting extracellular markers: BV510 anti-mouse CD45 (BioLegend, USA; 103138, 1:50), PE-CD11b (BioLegend, USA; 101208, 1:100), APC-CD11c (BioLegend, USA; 117310, 1:100), FITC anti-mouse I-A/I-E (BioLegend, USA; 107605, 1:200), PE/Cy7-CD103 (BioLegend, USA; 121425, 1:50), and Fixable Viability Dye eFluor 780 (eBioscience, USA; 65-0865-14, 1:100), followed by incubation at 4 °C for 30 min. The samples were subsequently analyzed via LSRFortessa flow cytometry (BD, USA), and the data were processed via FlowJo V10.8.1.

### Single-cell RNA sequencing (scRNAseq)

The raw FASTQ files were generated and demultiplexed via CeleScope RNA (v.3.0.1) from Singleron, followed by primary data analysis via CeleScope (v.1.10.0) via a custom reference package based on the Mus_musculus_ensembl_92 reference genome. Downstream data analysis was performed via Seurat v5.1.0.^37^ The cells were filtered on two criteria: (1) the number of detected genes per cell ranged from 200–6000, and (2) the proportion of mitochondrial gene counts (UMIs from mitochondrial genes/total UMIs) was less than 10%. The gene expression data were normalized via the SCTransform function in Seurat. The normalized SCT data were reduced to two dimensions via uniform manifold approximation and projection (UMAP) for visualization, with 30 principal components (PCs) as inputs. Differentially expressed genes (DEGs) were identified via the FindMarkers function for pairwise comparisons, with a log fold-change threshold of 0.25 applied for selection. Pseudotime trajectory analysis was performed with Monocle3,^38^ and cell‒cell communication analysis was conducted via CellChat.^39^

### Immunohistochemistry (IHC)

Fresh tissues removed from the mice were immediately fixed in 4% paraformaldehyde for 24 h, embedded in paraffin, and sectioned into 4 μm thick slices for immunohistochemical staining. After deparaffinization, the sections were subjected to antigen retrieval by boiling in Tris-EDTA (pH 9.0) solution for 20 min. After the sections cooled naturally to room temperature, they were incubated with 3% H_2_O_2_ for 10 min to block endogenous peroxidase activity. The sections were then blocked with normal goat serum at room temperature for 30 min, followed by incubation with the primary antibody overnight at 4 °C. After being washed with PBS, the sections were incubated with the secondary antibody at room temperature for 1 h, followed by color development via 3,3’-diaminobenzidine (DAB). Finally, the sections were counterstained with hematoxylin and scanned via a Pannoramic Midi digital slide scanning analysis system (3D Histech). The following primary antibodies were used: anti-cytokeratin 19 (Abcam, USA; ab52625, 1:1,000) and anti-adenovirus type 5 E1A (Abcam, USA; ab204123, 1:100).

### Tyramide signal amplification (TSA) multicolor immunohistochemistry

The sections were deparaffinized, subjected to antigen retrieval, endogenous peroxidase blocking, and blocking of nonspecific binding. During each staining cycle, the sections were incubated overnight at 4°C with different primary antibodies. The sections were subsequently incubated with HRP-labeled secondary antibodies at room temperature for 1 h, followed by staining with TSA fluorophores (AiFang Biological, China) for 8 min. The sections were placed in boiling antigen retrieval solution for 20 min to elute the antibodies. After the above steps were repeated, the sections were sequentially incubated with primary and secondary antibodies, followed by staining with TSA fluorophores (TYR-480, TYR-520, TYR-570, and TYR-690) in the specified order. Finally, the sections were stained with DAPI for 10 min and scanned via a Pannoramic Midi digital slide scanning system (3D Histech). The following primary antibodies were used: anti-CD8 alpha antibody (Abcam, USA; ab217344, 1:1000), anti-Granzyme B antibody (Abcam, USA; ab255598, 1:1000), anti-CD103 antibody (Abcam, USA; ab224202, 1:1000), anti-MHC class II antibody (Abcam, USA; ab23990, 1:500), CD69 rabbit PolymAb (ABclonal, China; A26620PM, 1:500), TCF1/TCF7 rabbit mAb (Cell Signaling Technology, USA; 2203, 1:500), and anti-CD8 alpha antibody (Abcam, USA; ab237709, 1:1000).

### Statistical analysis

Statistical analysis was performed via GraphPad Prism 10.1.2, with details and methods provided in the figure legends or main text. Two-tailed Student’s *t* test was used to analyze differences between two groups, whereas two-tailed one-way ANOVA or two-way ANOVA was used for comparisons among more than two groups. Survival curves were generated via Kaplan‒Meier estimates, and *p* values were calculated via the log-rank test. A *p* value of <0.05 was considered statistically significant. All the data were analyzed in a double-blinded manner by two statisticians.

## Supplementary information

Supplementary Materials including Figures, Figure legends and Tables

Supplementary Data include WES results of B16F10, MC38, Hep53.4 and mICCN-4 tumor

## Data Availability

All the data produced or analyzed in this study are provided within this published article and supplementary information files. The raw sequencing data have been deposited in the Genome Sequencing Archive database (GSA, https://ngdc.cncb.ac.cn/gsa/) under the accession number CRA028724 and CRA032496. The Cancer Genome Atlas (TCGA) datasets (CHOL and LIHC) referenced in this study are available from GEPIA (http://gepia.cancer-pku.cn/).
