# Supplementary material for: Oncolytic adenovirus delivery of neoantigens sensitizes low-mutation tumors to anti-PD-1 therapy and prevents metastasis
Source: Signal Transduct Target Ther. 2025 Dec 23;10:410. doi: 10.1038/s41392-025-02511-5 (PMC12722228; doi:10.1038/s41392-025-02511-5)
Supplement: Supplementary file 1 — Supplementary Materials including Figures, Figure legends and Tables [file 41392_2025_2511_MOESM1_ESM.docx]

**Supplementary Materials for**

Oncolytic adenovirus delivery of neoantigens sensitizes low-mutation tumors to anti-PD-1 therapy and prevents metastasis

Authors: Ke-Yu Shen*^,1,2^, Shi-Zhe Yu*^,1,2^, Ying-Han Su*^,#,1,2^, Sun-Zhe Xie ^1,2^, Chen Zhang^1,2^, Hao Xu^1,2^, SamI Yang^1^, Tian-Tian Zou^1^, Yan Fu^3^, Hao Wang^1^, Lin Fang^4^, Yan Zheng^5^, Chang-Qing Su^#,4,6^, Lun-Xiu Qin^#,1,2^

Correspondence to Lun-Xiu Qin (qinlx@fudan.edu.cn) & Ying-Han Su (yinghan_su@126.com) & Chang-Qing Su (suchangqing@gmail.com)

**This PDF file includes:**

Supplementary Figure 1-9 with their legends

Supplementary Table 1-3

**Other Supplementary Materials for this manuscript include the following:**

Data S1

**Supplementary Fig. 1** Neoantigen identification and oncolytic adenovirus vector construction. **a** Schematic representation of the construction of mouse intrahepatic cholangiocarcinoma cell line mICCN-4. **b** Microscopic view of mICCN-4. **c** Images of mICCN-4 subcutaneous and orthotopic tumors, including HE staining and CK19 immunohistochemistry (IHC) staining. **d-f** Mass spectrums of the MHC-I binding peptides detected by LC-MS/MS following immunoprecipitation. **g** Structural diagrams of the oncolytic adenovirus vector Ad5SVPF11 and the design of an oncolytic adenovirus expressing red fluorescence (DsRed). **h** Representative fluorescence images of tumor cell lines B16F10, MC38, Hep53.4, mICCN-4, and normal mouse liver cells AML12 infected with AdSVP-DsRed at 0, 48, and 96 hours. **i** Relative expression of E1A in these cell lines infected with AdSVP-DsRed at 0, 48, and 96 hours, as determined by qRT-PCR. **j** Relative expression of NAg in B16F10, MC38, Hep53.4 infected with AdSVP-NAg at 0, 48, and 96 hours determined by qRT-PCR assay. The data are representative of three independent experiments. The data were shown as the means ± SDs (**i, j**) and were representative of three (**h-j**) independent experiments.


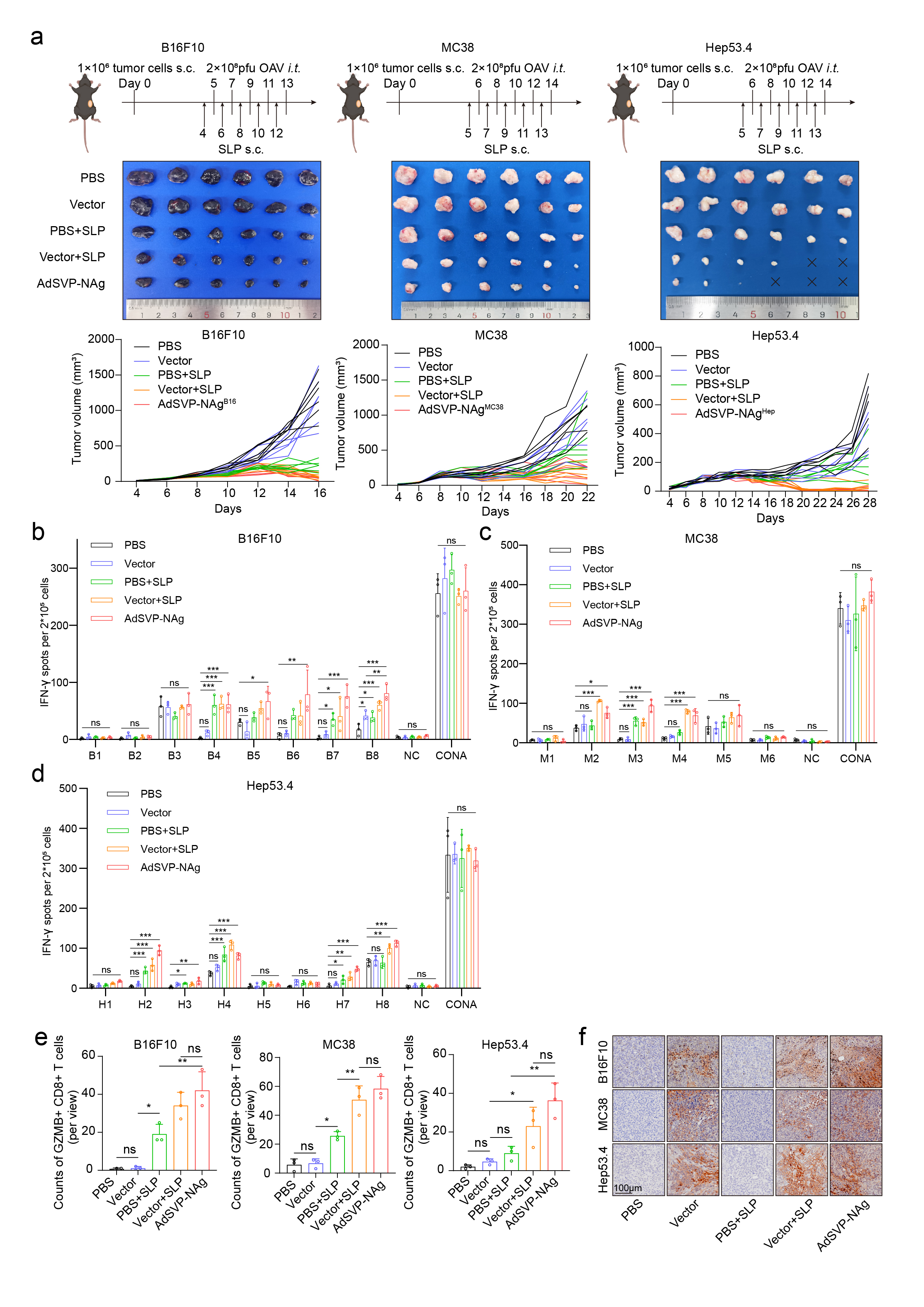


**Supplementary Fig. 2** Evaluation of the efficiency of oncolytic adenovirus delivery of neoantigens. **a** Top, experimental schematic. On Day 0, 1×10⁶ tumor cells were resuspended in 100 μL saline and subcutaneously injected into mice. For the B16F10 model, intratumoral injections of PBS or 2×10⁸ pfu OAV were administered every other day starting from Day 5, while subcutaneous injections of SLP were initiated on Day 4 and continued every other day for a total of five doses.​ For MC38 and Hep53.4 models, intratumoral injections of PBS or 2×10⁸ pfu OAV were administered every 2 days starting from Day 6, while subcutaneous injections of SLP were initiated on Day 5 and continued every 2 days for a total of five doses. ​Middle, tumor images at the end point. Bottom, tumor growth curves for individual mice. **b-d** Statistical plots for IFN-γ ELISpot results of B16F10 (**b**), MC38 (**c**) and Hep53.4 (**d**) model. **e** Counts of GZMB+ CD8+ T cells in immunofluorescence images. **f** Representative images of E1A IHC staining. The data were shown as the means ± SDs (**b, c, d, e**) and were representative of two (**a-f**) independent experiments. Significance was calculated by one-way ANOVA (**b-e**). *P<0.05, **P<0.01, ***P<0.001.


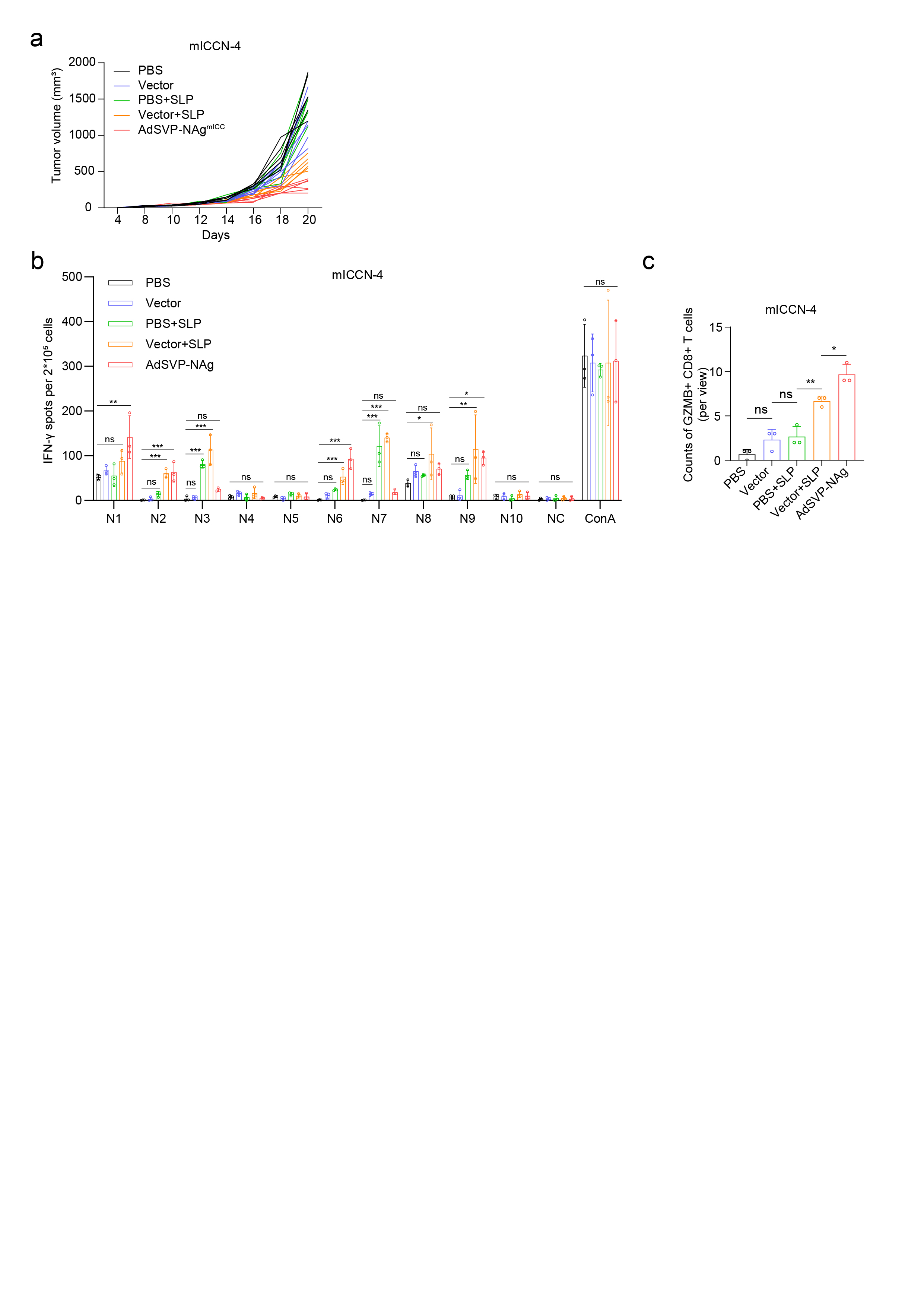


**Supplementary Fig. 3** Evaluation of the efficiency of oncolytic adenovirus delivery of neoantigens in mICCN-4. **a** Tumor growth curves for individual mice. **b** Statistical plots for IFN-γ ELISpot results of mICCN-4 model. **c** Statistical plots for counts of GZMB+ CD8+ T cells in immunofluorescence images. The data were shown as the means ± SDs (**b, c**) and were representative of two (**a-c**) independent experiments. Significance was calculated by one-way ANOVA (**b, c**). *P<0.05, **P<0.01, ***P<0.001. ns, no significance.


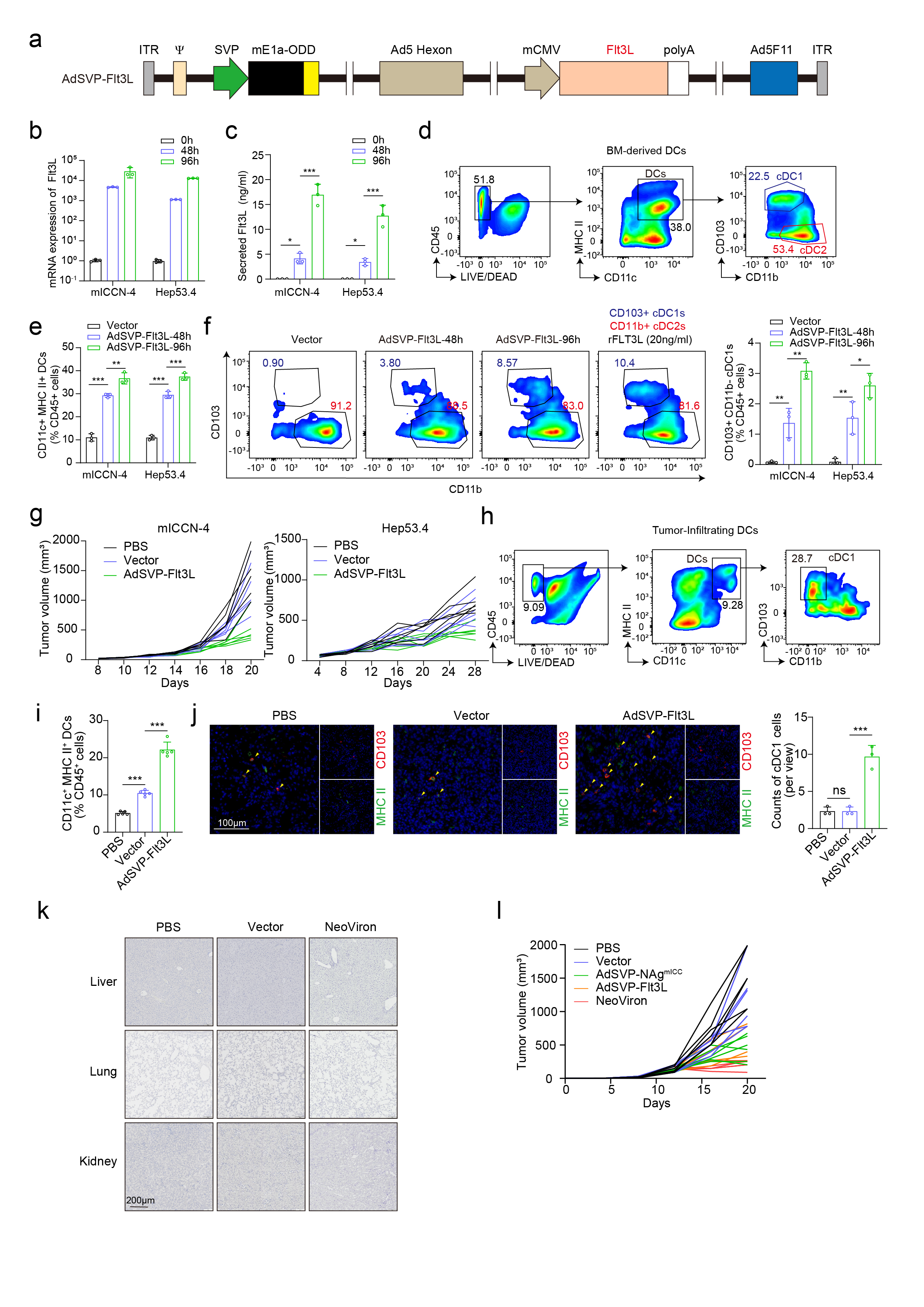


**Supplementary Fig. 4** In vivo experiment evaluating the efficiency of oncolytic adenovirus delivery of Flt3L. **a** Design of an oncolytic virus expressing Flt3L. **b, c** Relative mRNA expression (**b**) and concentration (**c**) of Flt3L in mICCN-4 and Hep53.4 infected with AdSVP-Flt3L at 0, 48, and 96 hours determined by qRT-PCR and ELISA, respectively. **d** Gating strategy for bone marrow-derived DCs. **e** Percentage of CD11c+ MHCII+ DCs. **f** Left, representative plots for bone marrow-derived DCs after co-culture. Recombinant Flt3L (rFL3L) was served as a control. Right, Percentage of CD103+ cDC1s. **g** Tumor growth curves for individual mice. **h** Gating strategy for DCs in tumor tissues. **i** Percentage of CD11c+ MHCII+ DCs in tumor tissues of mice inoculated with mICCN-4. **j** Immunofluorescence staining assays of CD103+ cDC1s in tumor tissues of mice inoculated with Hep53.4. **k** Healthy mice received tail vein injection of PBS, 2×10⁸ pfu Vector or NeoViron every other day for 3 times. IHC for E1A in liver, lung and kidney. **l** Tumor growth curves for individual mice. The data were shown as the means ± SDs (**b-f, i, j**) and were representative of two (**g-l**) or three (**b-f**) independent experiments. Significance was calculated by one-way ANOVA (**b-f, i, j**). **P*<0.05, ***P*<0.01, ****P*<0.001. ns, no significance.


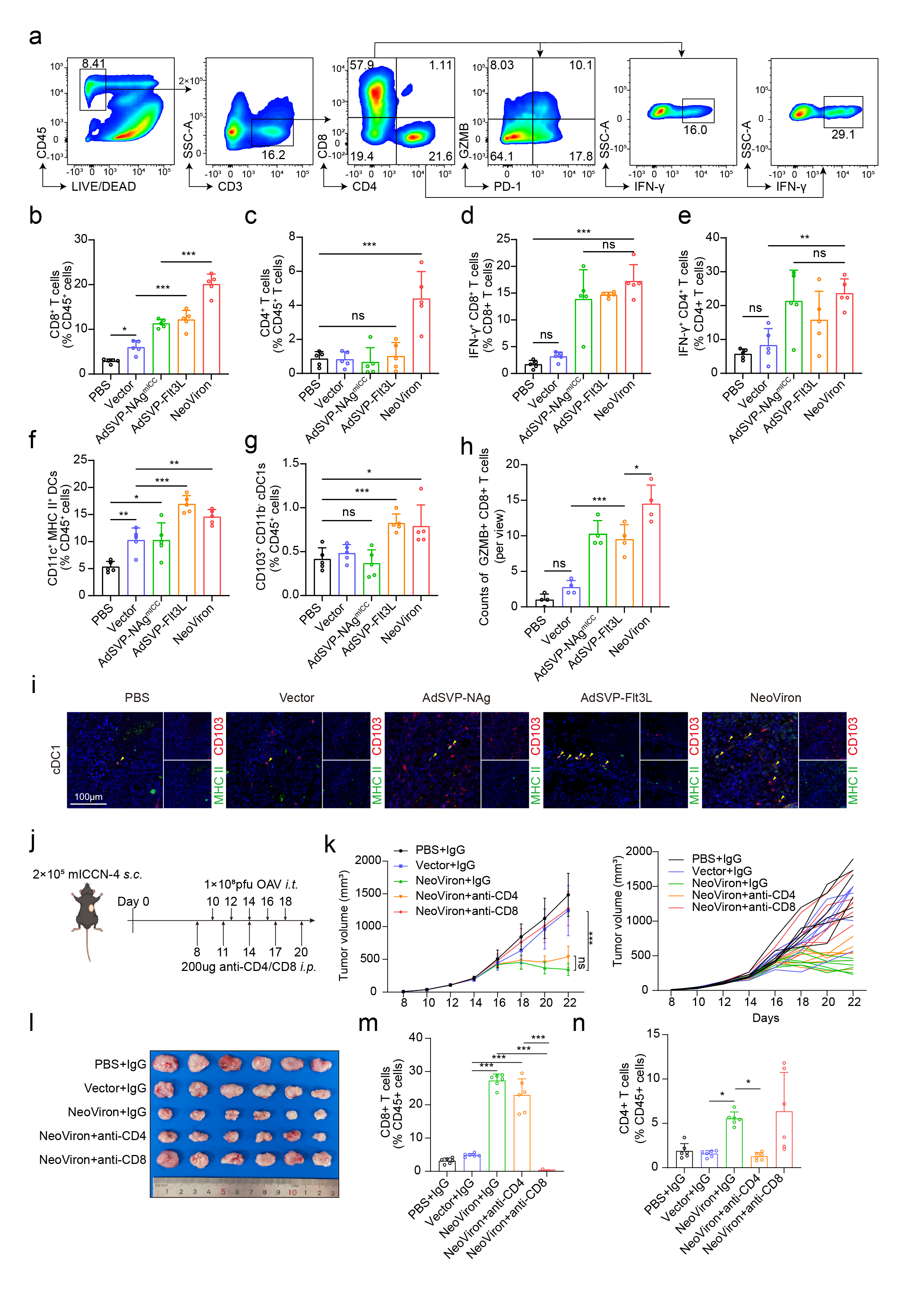


**Supplementary Fig. 5** In vivo validation of NeoViron. **a** Gating strategy for T cells. **b-i** C57BL/6 mice inoculated with mICCN-4 were treated with PBS, Vector, AdSVP-NAg, AdSVP-Flt3L or NeoViron. **b-g** Flow cytometric analysis of the percentage of intratumoral CD8+ T cells (**b**), CD4+ T cells (**c**), IFN-γ+ CD8+ T cells (**d**), IFN-γ+ CD4+ T cells (**e**), CD11c+ MHC II+ DCs (**f**) and CD103+ cDC1s (**g**). **h** Counts of GZMB+ CD8+ T cells detected by immunofluorescence staining assays. **i** Immunofluorescence staining assays of CD103+ cDC1s in tumor tissues. **j-n** On Day 0, 2×10^5^ tumor cells were resuspended in 100 μL saline and subcutaneously injected into mice. Intratumoral injections of PBS or 1×10⁸ pfu OAV were administered every other day starting from Day 10 for 5 doses, while intraperitoneal injections of 200 μg anti-CD4/CD8 were initiated on Day 8 and continued every three days. Experimental schematic (**j**), tumor growth curves (n=6 mice per group) (**k**), tumor images at the end point (**l**) and percentage of CD8+ T (**m**) and CD4+ T (**n**) infiltration in tumor tissues. The data were shown as the means ± SDs (**b-h, k, m, n**) and were representative of two (**b-n**) independent experiments. Significance was calculated by one-way ANOVA (**b-h, m, n**) or two-way ANOVA (**k**). **P*<0.05, ***P*<0.01, ****P*<0.001. ns, no significance.


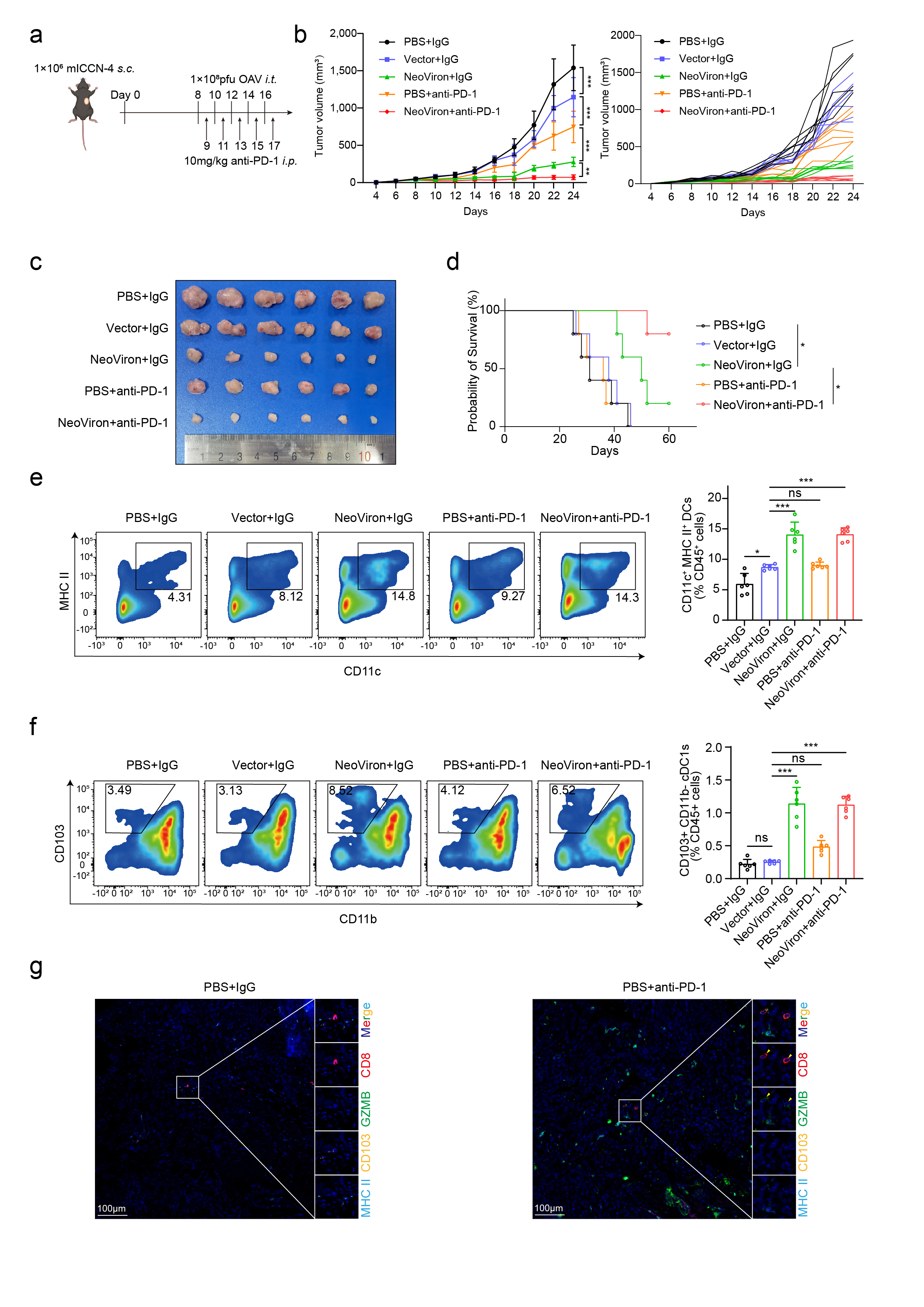


**Supplementary Fig. 6** NeoViron exhibits synergistic effects with anti-PD-1. **a-c** C57BL/6 mice inoculated with mICCN-4 were administered intratumoral injections starting from Day 8, with either PBS, 1×10⁸ pfu Vector, or NeoViron every other day for five doses. Concurrently, intraperitoneal injections of 10 mg/kg anti-PD-1 were initiated on Day 9 and administered every other day for five doses. Experimental schematic (**a**), tumor growth curves (n=6 mice per group) (**b**), and tumor images at the end point (**c**). **d-g** C57BL/6 mice with liver orthotopically implanted mICCN-4 were treated with PBS plus IgG, Vector plus IgG, NeoViron plus IgG, PBS plus anti-PD-1 or NeoViron plus anti-PD-1. Kaplan-Meier survival curve. (n = 6 mice per group) (**d**). Percentage of DCs (**e**) and cDC1s (**f**) infiltration in tumor tissues of each group. **g** Multiplex immunofluorescence images showing the colocalization of GZMB+ CD8+ T cells and CD103+ MHC II+ cDC1s. The data were shown as the means ± SDs (**b, e, f**) and were representative of two (**a-g**) independent experiments. Significance was calculated by one-way ANOVA (**e, f**), two-way ANOVA (**b**) or Log-rank Mantel-Cox test (**d**). **P*<0.05, ***P*<0.01, ****P*<0.001. ns, no significance.

**Supplementary Fig. 7** Combination therapy promotes the expansion of CD69+ CD8+ Trm and TCF1+ CD8+ Tstem cells. **a** Dot plot of the expression levels of marker genes for different cell clusters. **b** Top, UMAP visualization of CD4+ T cell subsets. Bottom left, dot plot of the expression levels of marker genes for CD4+ T cell subsets. Bottom right, alluvial plot showing the amounts of each CD4+ T cell subset across different groups. **c** Left, UMAP visualization of DC subsets. Middle, dot plot of the expression levels of marker genes for DC subsets. Right, alluvial plot showing the amounts of each DC subset across different groups. **d** Gene expression dynamics plots for Cd69, Tcf7, Gzmb, and Prf1 across CD8_T1_Gzmb, CD8_T2_ Tcf7, and CD8_T3_Cxcr4. **e** Circle plot depicting the MHC-I signaling pathway between cDC1 and each CD8+ T cell subset.

**Supplementary Fig. 8** Survival analysis reveal the impact of CD8+T, Trm and Tstem infiltration levels on OS and RFS. **a, b** The survival curves comparing OS and RFS between the high and low groups based on the median infiltration of T cell subtypes of huashan cohort. **a** CCA patient cohort. Top, the OS and RFS survival curves between the CD8 high and low groups. Middle, the OS survival curves between the Trm high and low groups. Bottom, the OS and RFS survival curves between the Tstem high and low groups. **b** HCC patient cohort. Top, the OS and RFS survival curves between the CD8 high and low groups. Middle, the OS survival curves between the Trm high and low groups. Bottom, the OS and RFS survival curves between the Tstem high and low groups. **c, d** The survival curves comparing OS and RFS between the high and low groups based on immune cell gene markers in the TCGA database. **c** CCA cohort. Top, the OS and RFS survival curves between the CD8 high and low groups. Middle, the OS and RFS survival curves between the Trm high and low groups. Bottom, the OS and RFS survival curves between the Tstem high and low groups. **d** HCC cohort. Top, the OS and RFS survival curves between the CD8 high and low groups. Middle, the OS and RFS survival curves between the Trm high and low groups. Bottom, the OS and RFS survival curves between the Tstem high and low groups. Log-rank Mantel-Cox test.


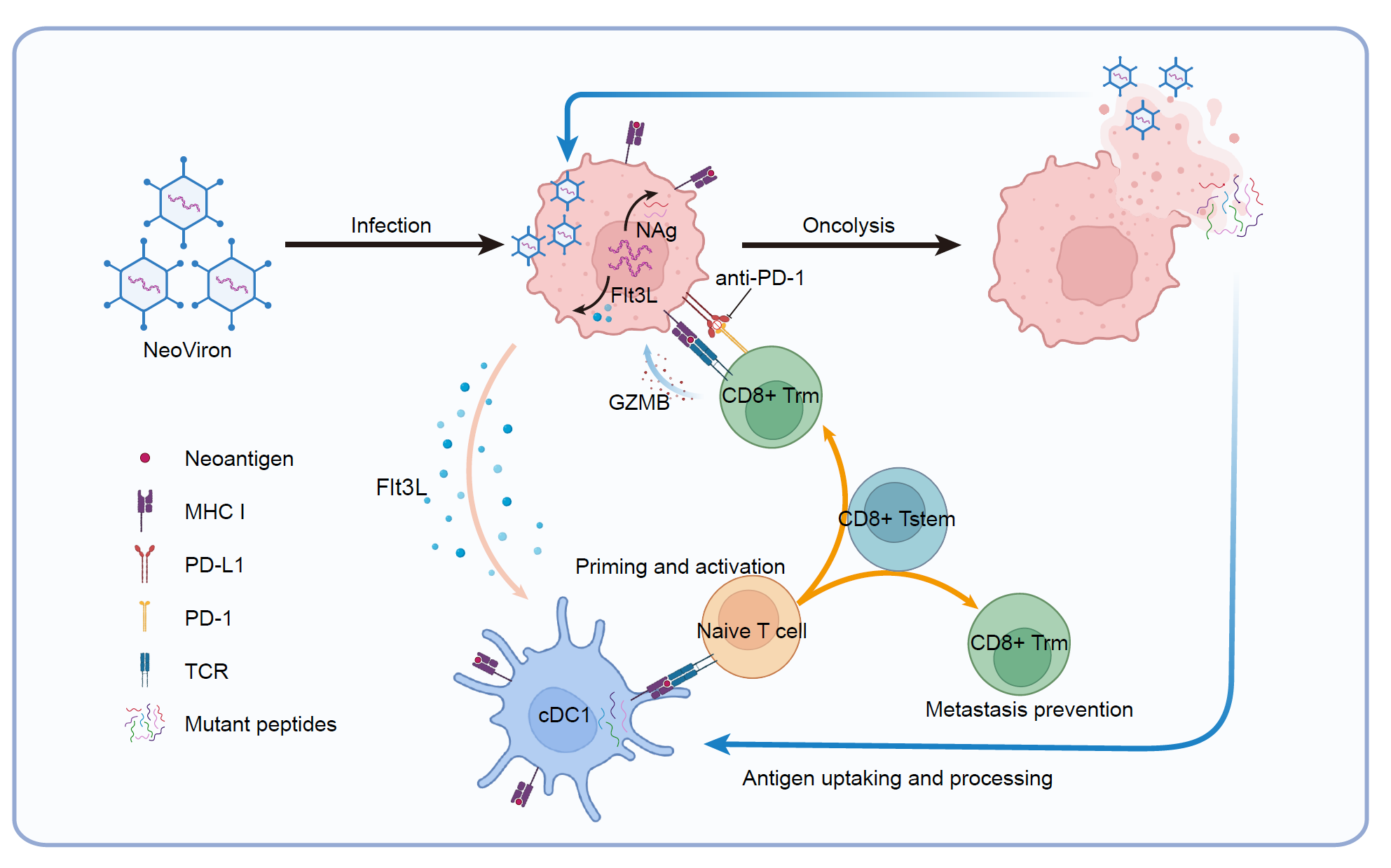


**Supplementary Fig. 9** Schematic diagram of the anti-tumor mechanism of NeoViron. This figure was created via resources from BioRender (www.biorender.com).

**Supplementary Tables**

Supplementary Table 1. The list of neoantigen sequences identified by WES combined with MS in B16F10, MC38, and Hep53.4.

| **Tumor** | **ID** | **Gene** | **Mutation** | **Epitope** | **Length** | **H-2Db_Rank** | **H-2Kb_Rank** | **Predicted_BindLevel** | **Extended_Epitope** |
| --- | --- | --- | --- | --- | --- | --- | --- | --- | --- |
| B16F10 | B1 | Usp15 | Y578H | KLHNLLLLR | 9 | 62.6667 | 47.5926 | - | FLMAIPRNNTEDKLHNLLLLRMCRYVKMS |
|  | B2 | Ap5b1 | K624T | LSHLSSSTL | 9 | 0.6744 | 1.3835 | WB | ARLYYVLLSHLSSSTLGMALGPSLAAPAL |
|  | B3 | Rapgef2 | L471P | KEPLTRLS | 8 | 43.6667 | 47.2593 | - | LSITVKTNLFVFKEPLTRLSEEKRNGAPH |
|  | B4 | Vmn2r16 | Q418P | ESPKMPKL | 8 | 1.8651 | 0.8036 | WB | AHTYHELILQQVESPKMPKLKGVFSDCHQ |
|  | B5 | Speer4d | P63T | QNTLYEKL | 8 | 3.5874 | 0.8209 | WB | TEESMNKRPYHRQNTLYEKLKLKEKEIMT |
|  | B6 | Dnmt1 | E553G | TGDSLLRHA | 9 | 7.8634 | 12.8421 | - | TTVPPSTINVNRFTGDSLLRHAQFVVSQV |
|  | B7 | Nbea | K2238N | VNKVVYSL | 8 | 2.2272 | 0.3923 | SB | RTSVMFNFPDQATVNKVVYSLPRVGVGTS |
|  | B8 | Ndufs6 | V4A | AALTFRRL | 8 | 0.0661 | 0.0059 | SB | MAAALTFRRLLTLPRAAR |
| MC38 | M1 | Cdc23 | A195P | VEPTHVLPL | 9 | 0.5216 | 1.5359 | WB | KLDLVKEAIDVFVEPTHVLPLHWGAWLEL |
|  | M2 | Ttn | R19795H | VTNNCNHL | 8 | 6.2682 | 2.4419 | - | ASKKAWVSVTNNCNHLSYKVTNLQEGAIY |
|  | M3 | Ap4b1 | V277F | VRFKGPLL | 8 | 25.6222 | 0.8983 | WB | AKKFPHVQTDVLVRFKGPLLAACSSESRE |
|  | M4 | Cplane2 | L140H | NADAFHFL | 8 | 8.3095 | 1.9616 | WB | DHMLPACKENADAFHFLFSFTDRASFEDL |
|  | M5 | Anpep | I238N | TLNYPNNL | 8 | 39.2917 | 2.6127 | - | FDEPAMKAMFNITLNYPNNLIALSNMLPK |
|  | M6 | Hydin | L4282P | ELSGPKPLL | 9 | 21.5177 | 14.1702 | - | KFNFSYQAELSGPKPLLQYLDFTPTDSSV |
| Hep53.4 | H1 | Phldb3 | V119M | LEALTRMAL | 9 | 12.1901 | 14.6529 |  | ARRLRGQQLEALTRMALMEQRVKELQRQK |
|  | H2 | Vmn2r75 | M781K | KLLTFSKL | 8 | 16.8602 | 0.0975 | SB | LPDTFNEAKLLTFSKLVFCSVWITFIPVY |
|  | H3 | Vmn2r75 | M781K | AKLLTFSK | 8 | 63.125 | 31.9254 | - | LPDTFNEAKLLTFSKLVFCSVWITFIPVY |
|  | H4 | Vmn2r75 | M781K | KLLTFSKLV | 9 | 10.9654 | 2.5314 | - | LPDTFNEAKLLTFSKLVFCSVWITFIPVY |
|  | H5 | Vmn2r75 | M781K | AKLLTFSKL | 9 | 3.5365 | 0.9594 | WB | LPDTFNEAKLLTFSKLVFCSVWITFIPVY |
|  | H6 | Vmn2r75 | M781K | AKLLTFSKLV | 10 | 22.5469 | 18.1104 | - | LPDTFNEAKLLTFSKLVFCSVWITFIPVY |
|  | H7 | Fcmr | T204S | SASKSPAL | 8 | 7.7205 | 1.8229 | WB | TQHPRVYRAFSVSASKSPALLPATTASKT |
|  | H8 | Abca13 | T2090N | TNVQLPQL | 8 | 13.7616 | 0.2762 | SB | DINKNIQGAGLQNTNVQLPQLLELLDSSP |

Supplementary Table 2. The list of neoantigen sequences predicted by NetMHCPan4.1 based on WES in mICCN-4.

| **ID** | **Gene** | **Mutation** | **Neoepitope** | **Length** | **H-2Db_Rank** | **H-2Kb_Rank** | **Predicted_BindLevel** | **Extended_Epitope** |
| --- | --- | --- | --- | --- | --- | --- | --- | --- |
| N1 | F2rl1 | C163R | RSILFMTCL | 9 | 0.4811 | 0.3185 | SB | LCKVLIGFFYGNMYRSILFMTCLSVQRYW |
| N2 | Nme3 | V20L | YSGLNERTFL | 10 | 0.1062 | 15.9552 | SB | LTIFANLFPSAYSGLNERTFLAVKPDGVQ |
| N3 | Kcnb2 | L885V | YKMENHVFA | 9 | 0.3387 | 16.6613 | SB | KKDSSQEGYKMENHVFAPEIHSNPGDTGH |
| N4 | Nme3 | V20L | SGLNERTFL | 9 | 0.3673 | 2.483 | SB | LTIFANLFPSAYSGLNERTFLAVKPDGVQ |
| N5 | Elavl2 | A361V | AVMAIASL | 8 | 10.5274 | 0.2365 | SB | KGFGFVTMTNYDEAVMAIASLNGYRLGDR |
| N6 | Elavl2 | A361V | VTMTNYDEAV | 10 | 0.2528 | 11.0281 | SB | KGFGFVTMTNYDEAVMAIASLNGYRLGDR |
| N7 | Abcg2 | I514T | IMMFTLTM | 8 | 5.2079 | 0.4765 | SB | KKTVDAFFIMMFTLTMVAYTASSMALAIA |
| N8 | Olfr135 | P26S | LSLFIILL | 8 | 16.9319 | 0.7504 | WB | FILLGFADRPWLELSLFIILLVTYPTAMI |
| N9 | Abcg2 | I514T | MMFTLTMV | 8 | 12.5456 | 0.856 | WB | KKTVDAFFIMMFTLTMVAYTASSMALAIA |
| N10 | Insrr | M1159L | KAPESLKDGI | 10 | 1.0976 | 16.0732 | WB | YYRKGGKGLLPVRWKAPESLKDGIFTTHS |

Supplementary Table 3. The list of synthetic peptides used for ELISpot assays and SLP vaccination

| **ID** | **Gene** | **Epitope** | **SLP** |
| --- | --- | --- | --- |
| B1 | Usp15 | KLHNLLLLR | FLMAIPRNNTEDKLHNLLLLRMCRYVKMS |
| B2 | Ap5b1 | LSHLSSSTL | ARLYYVLLSHLSSSTLGMALGPSLAAPAL |
| B3 | Rapgef2 | KEPLTRLS | LSITVKTNLFVFKEPLTRLSEEKRNGAPH |
| B4 | Vmn2r16 | ESPKMPKL | AHTYHELILQQVESPKMPKLKGVFSDCHQ |
| B5 | Speer4d | QNTLYEKL | TEESMNKRPYHRQNTLYEKLKLKEKEIMT |
| B6 | Dnmt1 | TGDSLLRHA | TTVPPSTINVNRFTGDSLLRHAQFVVSQV |
| B7 | Nbea | VNKVVYSL | RTSVMFNFPDQATVNKVVYSLPRVGVGTS |
| B8 | Ndufs6 | AALTFRRL | MAAALTFRRLLTLPRAAR |
| M1 | Cdc23 | VEPTHVLPL | KLDLVKEAIDVFVEPTHVLPLHWGAWLEL |
| M2 | Ttn | VTNNCNHL | ASKKAWVSVTNNCNHLSYKVTNLQEGAIY |
| M3 | Ap4b1 | VRFKGPLL | AKKFPHVQTDVLVRFKGPLLAACSSESRE |
| M4 | Cplane2 | NADAFHFL | DHMLPACKENADAFHFLFSFTDRASFEDL |
| M5 | Anpep | TLNYPNNL | FDEPAMKAMFNITLNYPNNLIALSNMLPK |
| M6 | Hydin | ELSGPKPLL | KFNFSYQAELSGPKPLLQYLDFTPTDSSV |
| H1 | Phldb3 | LEALTRMAL | ARRLRGQQLEALTRMALMEQRVKELQRQK |
| H2 | Vmn2r75 | KLLTFSKL | LPDTFNEAKLLTFSKLVFCSVWITFIPVY |
| H3 | Vmn2r75 | AKLLTFSK | LPDTFNEAKLLTFSKLVFCSVWITFIPVY |
| H4 | Vmn2r75 | KLLTFSKLV | LPDTFNEAKLLTFSKLVFCSVWITFIPVY |
| H5 | Vmn2r75 | AKLLTFSKL | LPDTFNEAKLLTFSKLVFCSVWITFIPVY |
| H6 | Vmn2r75 | AKLLTFSKLV | LPDTFNEAKLLTFSKLVFCSVWITFIPVY |
| H7 | Fcmr | SASKSPAL | TQHPRVYRAFSVSASKSPALLPATTASKT |
| H8 | Abca13 | TNVQLPQL | DINKNIQGAGLQNTNVQLPQLLELLDSSP |
| N1 | F2rl1 | RSILFMTCL | LCKVLIGFFYGNMYRSILFMTCLSVQRYW |
| N2 | Nme3 | YSGLNERTFL | LTIFANLFPSAYSGLNERTFLAVKPDGVQ |
| N3 | Kcnb2 | YKMENHVFA | KKDSSQEGYKMENHVFAPEIHSNPGDTGH |
| N4 | Nme3 | SGLNERTFL | LTIFANLFPSAYSGLNERTFLAVKPDGVQ |
| N5 | Elavl2 | AVMAIASL | KGFGFVTMTNYDEAVMAIASLNGYRLGDR |
| N6 | Elavl2 | VTMTNYDEAV | KGFGFVTMTNYDEAVMAIASLNGYRLGDR |
| N7 | Abcg2 | IMMFTLTM | KKTVDAFFIMMFTLTMVAYTASSMALAIA |
| N8 | Olfr135 | LSLFIILL | FILLGFADRPWLELSLFIILLVTYPTAMI |
| N9 | Abcg2 | MMFTLTMV | KKTVDAFFIMMFTLTMVAYTASSMALAIA |
| N10 | Insrr | KAPESLKDGI | YYRKGGKGLLPVRWKAPESLKDGIFTTHS |

**Supplementary Data**

**Data S1.** WES results of B16F10, MC38, Hep53.4 and mICCN-4 tumor.
